# Stereoselective RNA reaction with chiral 2′-OH acylating agents†

**DOI:** 10.1039/d3sc03067a

**Published:** 2023-11-07

**Authors:** Ryuta Shioi, Lu Xiao, Sayantan Chatterjee, Eric T. Kool

**Affiliations:** a Department of Chemistry, Stanford University Stanford CA 94305 USA kool@stanford.edu; b Sarafan ChEM-H, Stanford University Stanford CA 94305 USA

## Abstract

The reactivity of RNA 2′-OH groups with acylating agents has recently been investigated for high-yield conjugation of RNA strands. To date, only achiral molecules have been studied for this reaction, despite the complex chiral structure of RNA. Here we prepare a set of chiral acylimidazoles and study their stereoselectivity in RNA reactions. Reactions performed with unfolded and folded RNAs reveal that positional selectivity and reactivity vary widely with local RNA macro-chirality. We further document remarkable effects of chirality on reagent reactivity, identifying an asymmetric reagent with 1000-fold greater reactivity than prior achiral reagents. In addition, we identify a chiral compound with higher RNA structural selectivity than any previously reported RNA-mapping species. Further, azide-containing homologs of a chiral dimethylalanine reagent were synthesized and applied to local RNA labeling, displaying 92% yield and 16 : 1 diastereoselectivity. The results establish that reagent stereochemistry and chiral RNA structure are critical elements of small molecule-RNA reactions, and demonstrate new chemical strategies for selective RNA modification and probing.

## Abstract 2

The 2′-OH groups of RNA are shown to react differentially with opposite enantiomers of chiral acylating agents, with applications in conjugation and mapping of the biomolecule.

## Graphical abstract

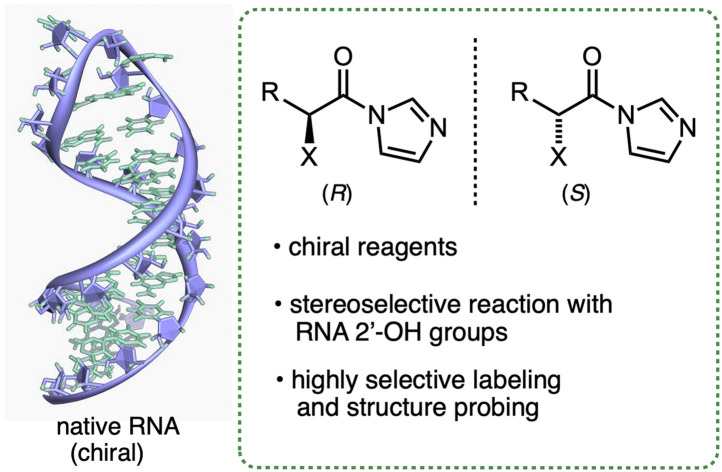

## Introduction

**Fig. 1:**
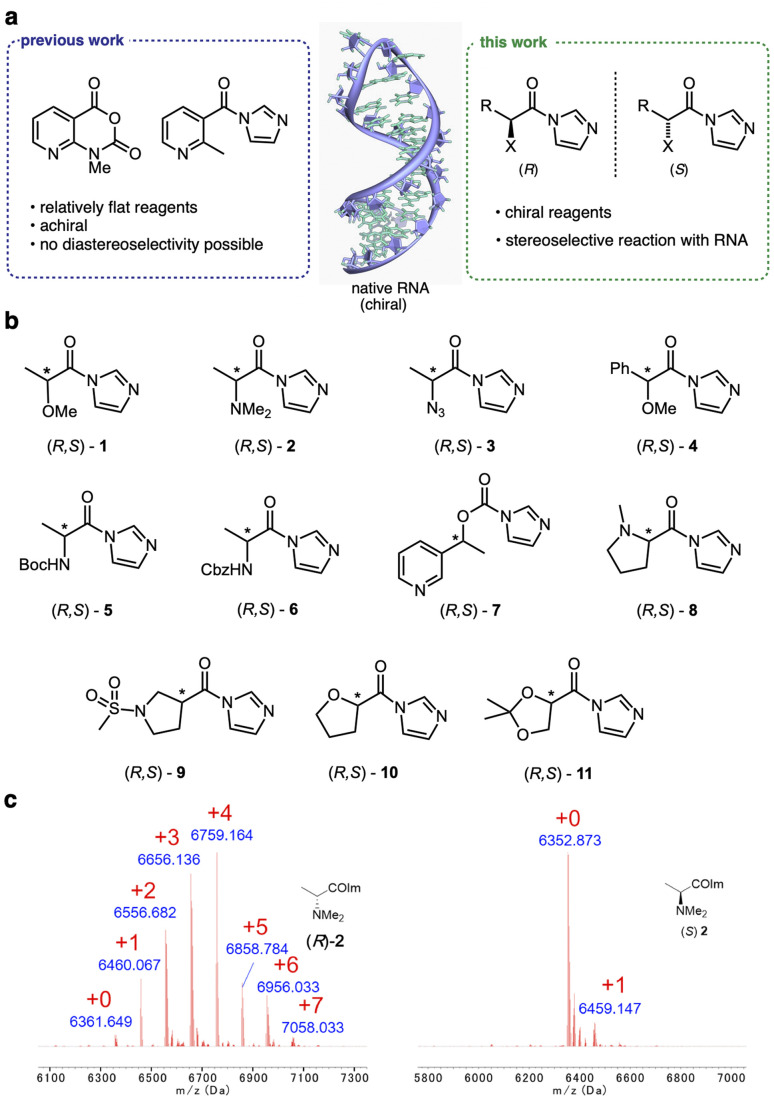
Chiral reagents exhibit stereoselective acylation of 2′-OH hydroxyl groups in RNA. (a) Recently reported reagents reacting with RNA 2′-OH groups have been achiral and relatively flat. New reagents possess asymmetric centers, conferring stereoselectivity (Table 1). Heteroatoms (X) are included to enhance solubility and carbonyl electrophilicity. R denotes varied alkyl and aryl substitution. (b) Structures of chiral acylimidazole enantiopairs screened for diastereoselective reaction with RNA. Asterisks mark asymmetric centers. (c) MALDI-TOF mass spectra of the products of reaction of (*R*)-2 and (*S*)-2 (100 μM, 2 h, 0 °C) with a single-stranded RNA, showing up to five substitutions and high conversion for the (*R*) enantiomer, but only one substitution and low conversion for the (*S*) isomer. Red numerals denote number of adducts on the RNA; “+0” indicates unmodified starting RNA mass peak.

The nucleophilic reactivity of RNA 2′-OH groups has proven broadly useful in probing, labeling, and conjugating RNA.^1–3^ The 2′-OH group as a reactive handle offers the advantage of near-universal availability at virtually every position of RNA. Selective control of reactions at specific sites along the RNA enables functionalization of both small and large RNAs for future biotechnological and biomedical applications.^4,5^ For high-yield preparative reactions, acyl small-molecule reagents (Fig. 1) have been developed for substituting RNA with useful moieties such as biotin,^6,7^ fluorophores,^4,8^ or click handles.^8,9^ Reactions can be carried out at super-stoichiometric levels, reacting stochastically at multiple sites along an RNA strand,^10,11^ or they can be directed locally for high-yield reactions by inducing the formation of small reactive loops.^4,5,12^ Superstoichiometric reactions can enable control of RNA functions^13–15^ and protect RNA from degradation.^16^ Site-localized reactions have been employed for control of translation and for site-specific labeling.^4,5^ A challenge for developing site-selective RNA reactions is the problem of the existence of many chemically similar hydroxyls that all have the potential to react. For this reason, identifying mechanisms for altering or controlling selectivity remains an important avenue of investigation.

RNA can fold into complex three-dimensional shapes, and is inherently chiral due to the (d)-ribose sugars that comprise the phosphodiester backbone. Chirality in nucleic acids is widely discussed and has been leveraged in numerous studies for selective recognition of small molecules with DNA;^17–21^ for example, chiral metal intercalator complexes have long been studied for their selectivity in associating with right-handed B-DNA helices *versus* left-handed structures.^17^ However, for RNA much less attention has been paid to chiral recognition by small molecules.^22^ A chief example has been the testing of RNA on solid support as a stationary phase for chiral chromatography.^23,24^ Chiral fluorinated diamines have also been tested as NMR probes of RNA.^25^ RNA aptamers selected for binding a naturally occurring small molecule have in a few cases also been shown to be selective for that enantiomer over the opposite one.^26,27^ While the number of small molecule ligands known to bind RNA is growing rapidly, the R-BIND database^28^ lists no enantiomer pairs among 131 ligands. However, in a recent example of chiral recognition by synthetic compounds, ligands for a bacterial RNA riboswitch have been shown to be stereoselective in binding.^29^

Although large oligonucleotide conjugates have been investigated more recently,^30–32^ a small number of early studies described reactions of nucleotides with chiral small molecules. Prebiotic chemistry experiments several decades ago tested the reactions of activated amino acids with dinucleotides in DMF solutions, and documented yields of up to 12% at internal 2′-OH and stereoselectivity up to 2 : 1.^33^ One report also tested reactions with homopolymer RNAs, noting yields of 7% in reaction of polyU (for example) with an alanine derivative.^34,35^ As solid-phase RNA/DNA synthesis was not yet available, no defined RNA sequences or folded contexts were tested. Thus it remained unknown whether preparatively useful yields could be achieved with chiral compounds, whether higher levels of selectivity are possible, and whether folded RNA structure might affect stereoselectivity. Given this lack of information, multiple questions arise: to what degree can reagent stereochemistry influence reactions at 2′-OH? Second, does the addition of steric bulk that confers asymmetry near the reactive carbonyl hinder RNA reaction? Finally, and importantly, how does RNA folded structure locally influence diastereoselectivity?

To begin to address these questions, we report here the study of enantiopairs of aqueous-soluble acylimidazole reagents having varied asymmetric substitution near their reactive carbonyl groups. Our findings show that with appropriate substitution, reagents can demonstrate high diastereoselectivity and preparatively useful yields in RNA reaction. The results establish new strategies for selective RNA recognition, with applications in conjugation, modification, and mapping of the biopolymer.

## Results and discussion

### Reagent design and synthesis

To initiate a survey of stereoselectivity, we selected a range of small chiral carboxylic acid precursors for which both enantiomers are readily available (Fig. 1). Structures contained α- or β-heteroatoms (O,N) to enhance aqueous solubility. Carboxylic acids were converted to activated form with carbonyldiimidazole in anhydrous DMSO, directly producing solutions of the reagents for testing (see details in the ESI file†).

### Screening for reagent selectivity

**Fig. 2:**
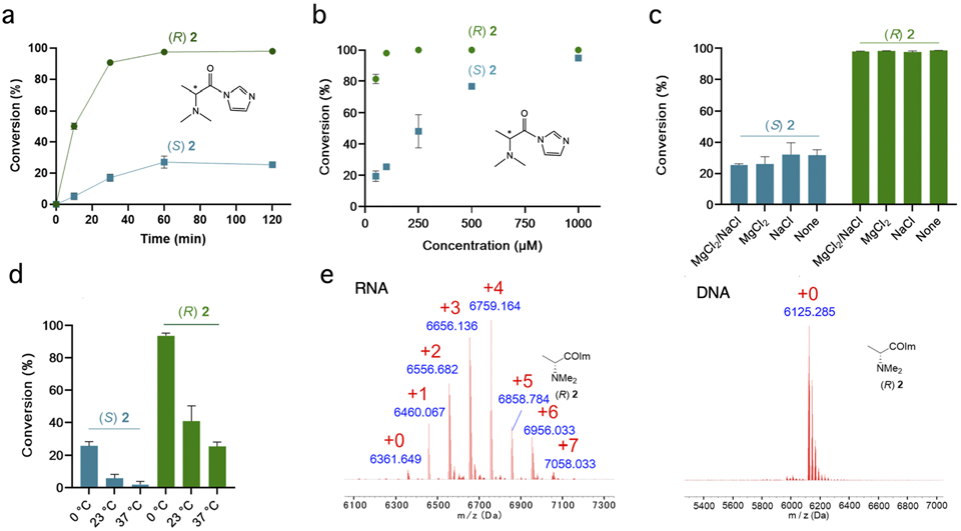
Characterizing diastereoselectivity and conditions for RNA reactions of chiral reagents (*R*,*S*)-2. (a) Diastereoselectivity of chiral amine scaffold (*R*,*S*)-2 as a function of time, reacting with 20nt ssRNA. Conditions: 10 μM RNA, 100 μM reagent, MOPS buffer pH 7.5, 0 °C (note: zero conversion at zero time point is not measured but assumed). (b) Effect of reagent concentrations (50–1000 μM) on conversion and selectivity at 2 h time point (0 °C). Selectivity is 3.9 : 1 at 100 μM averaged over the RNA strand. (c) Salts added to reaction (6 mM MgCl_2_ or 100 mM NaCl) have little effect on stereoselectivity (“none” indicates MOPS buffer only); (d) Effect of reaction temperature on conversion and diastereoselectivity. Conditions were the same as (a) (2 h time point) except varied temperature. (e) Comparison of MALDI-TOF data for reagent (*R*)-2 reacting with ssRNA (calc *m*/*z* 6352) and ssDNA (*m*/*z* 6116) of the same sequence (conditions as in (a), 2 h time point), showing little or no reaction with DNA. This confirms the 2′-OH group as the reaction site in RNA. Data in a-d are averages of 3 replicates, ±s.d.

Initial screening of RNA reactions was carried out using reagents in Na^+^/Mg^2+^/MOPS buffer (pH 7.5) with 20% DMSO at 0 °C for 2 h, using a target single-stranded 20 nucleotide (nt) RNA of mixed sequence. Yields of adducts were assessed by MALDI-TOF mass spectrometry (see examples in Fig. 1, 2 and S1†), assessing the conversion of the unmodified RNA to higher-mass products containing one or more acyl groups, and differential yields were recorded (Table 1). As stereoselectivity varies with time and reaction conversion (see below), reagent concentrations for each pair were adjusted for this initial screening to roughly similar levels of conversion.

**Table 1:** Reaction conversion and diastereoselectivity for screening chiral scaffords 1–11 during acylation of single-stranded RNA^a^

Compound	Conversion^b^ (%)	Diastereoselectivity^c^ (*R/S*)
(*R*) 1^d^	57	0.61
(*S*) 1^d^	94	
(*R*) 2^e^	98	3.92
(*S*) 2^e^	25	
(*R*) 3	55	1.34
(*S*) 3	41	
(*R*) 4^f^	56	1.16
(*S*) 4^f^	48	
(*R*) 5^g^	77	0.86
(*S*) 5^g^	90	
(*R*) 6	57	0.83
(*S*) 6	69	
(*R*) 7^h^	20	1.11
(*S*) 7^h^	18	
(*R*) 8	38	2.00
(*S*) 8	19	
(*R*) 9^i^	67	1.63
(*S*) 9^i^	41	
(*R*) 10^j^	72	0.78
(*S*) 10^j^	92	
(*R*) 11	41	0.51
(*S*) 11	80	

a Conditions: RNA acylation yields for the chiral acylating reagents with a single-stranded RNA. 10 μM RNA was treated with 200 mM reagent, 20% DMSO in water for 2 h at 0 °C.

b Conversion of starting RNA to acylated products (at 2 h) as measured by MALDI-TOF MS.

c This is an aggregate ratio of all reactive sites in the test RNA.

d 80 mM reagent was used.

e 100 μM reagent.

f 50 mM reagent.

g 100 mM reagent.

h 200 mM reagent, 6 h.

i 1 mM reagent.

j 20 mM reagent.

Among these chiral scaffolds, we observed that several yielded significant levels of diastereoselectivity in reaction with RNA (Table 1, Fig. S1 and S2†). Note that the reaction sites in the single-stranded RNA were not controlled, and thus conversions reflect aggregate yields, and diastereoselectivities represent aggregate selectivity in esterification reactions across these multiple 2′-OH sites. Highest diastereoselectivity in this initial screen was observed for compounds 2, 8, and 11, with selectivities of up to 3.9 : 1 (*R* : *S*) for alanine derivative 2, and 2 : 1 for structurally related proline derivative 8. Interestingly, the stereochemical preference varied, with α-amino species showing preference for the *R* isomer (*e.g.*2, 3, 8), while others (primarily α-alkoxy compounds such as 1, 10, 11) reacted selectively as the *S* enantiomer.

### Effect of reaction conditions

We next examined the most selective scaffold in the initial survey, (*R*,*S*)-*N*,*N*-dimethylalanine imidazolide (2), in more detail by investigating the effects of time and temperature on yields (Fig. 2). Fig. 2a shows near complete (>90%) conversion with the single-stranded 20 nt RNA in 1 h for the (*R*) enantiomer, but its mirror image achieves only <20% conversion even at extended times. Diastereoselectivity (dr) was 10 : 1 at 50% conversion and 5.3 : 1 at 90% conversion. The (*R*) enantiomer of 2 reacts at remarkably low concentrations (Fig. 2b), with near-complete conversion at 100 μM, much lower than previous reagents for stoichiometric RNA reactions.^8,10^ Its half-life in water (4.8 min; Fig. S3†) is not exceptionally short as compared with other RNA-acylating species.^8,9^ Comparison with a dimethylglycine analogue lacking the α-methyl group^16^ (Fig. S4†) suggests that the methyl group that confers chirality to (*R*)-2 also unexpectedly increases its reactivity with RNA.

Cations in solution can strongly affect RNA dynamics;^36^ however, we found that the presence of 100 mM Na^+^ or 6 mM Mg^2+^ in solution had no effect on reaction conversions or stereoselectivity of 2 with single-stranded RNA (Fig. 2c). We also investigated the effect of temperature on reactions (Fig. 2d), and observed increasing diastereoselectivity but decreasing reaction conversions with higher temperature.

Comparisons of reactions with RNA and DNA of the same sequence (Fig. 2e and S5†) ruled out significant reaction at exocyclic amines of the nucleobases.^37^ The results indicate that, as with most acylimidazoles tested to date, reaction occurs essentially exclusively at 2′-OH groups, yielding ester linkages. Even the primary 5′-alcohol of DNA does not appear to react substantially, consistent with the observation that the increased acidity of 2′-OH groups is important for promoting reaction with RNA.^38^ As an additional control for observations of stereoselectivity, we confirmed that the two enantiomers of the most selective scaffold (*R*,*S*)-2 react equally with an achiral alcohol (Fig. S6†).

For an initial test of whether RNA folded structure can influence diastereoselectivity of 2′-OH acylation, we compared reactions of acylimidazole scaffolds 1 and 2 with three RNAs: single-stranded RNA, a hairpin containing a 3 nt loop, and a hairpin containing a small 1-nt bulge loop (Fig. S7 and Table S1†). Diastereoselectivity (as measured in aggregate over multiple reaction sites) was not markedly different over the three contexts; again, the methoxy species displayed the opposite chiral preference ((*S*) favored) as compared with the dimethylamino compounds ((*R*) favored).

### Probing chiral selectivity over a range of folded RNA structures

**Fig. 3:**
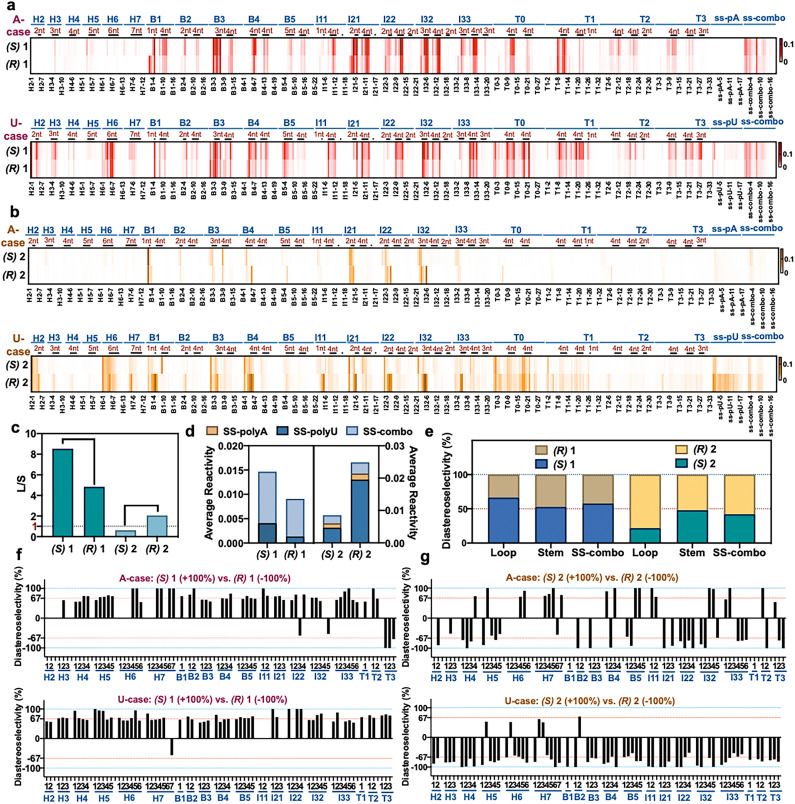
The effect of varied RNA folded structure on local reactivity and stereoselectivity of chiral reagents. (a and b) Relative reactivity of (*R*) and (*S*) enantiomers of 1 and 2 at each position within a library of folded RNAs^12^ (native (d) configuration), measured by deep sequencing. 884 nucleotides of sequence and 20 loop motifs are represented in the library. Loops are marked by dark bars and denoted by size, and stems fall in between. Sequences are as published;^12^ H = hairpin loop; B = bulge loop; I = internal loop; T = three-way junction loop, with numbers indicating loop size. Conditions: 50 mM 1 or 100 μM 2, 0 °C, 2 h. (c) Dynamic range of reactivity as measured by loop/stem (L/S) reactivity ratios for two enantiopairs of reagents. (d) Diastereoselectivity of reagents (*R*,*S*)-1 and (*R*,*S*)-2 with three single-stranded RNAs (SS-polyA, SS-polyU, mixed-sequence SS-combo). (e) Diastereoselectivity of reagents (*R*,*S*)-1 and (*R*,*S*)-2 averaged over all loops, all double-stranded stems, and for a single-stranded RNA (ss-combo) containing all bases. (f and g) Plots of diastereoselectivity at each position of loop nucleotides in the 43 RNAs, as measured by differential *R*/*S* reactivity of reagent 1 (f) and 2 (g).

The above experiments measured conversion yields in aggregate over multiple 2′-OH positions, and thus the selectivity at specific sites remained unknown. This prompted the question of how selectivity and reactivity at each local 2′-OH can vary depending on diverse folded RNA structural contexts. To evaluate this, we selected two enantiopairs of reagents that exhibited relatively high selectivity in the initial screening, compounds (*R*,*S*)-1 and (*R*,*S*)-2, and reacted the four compounds individually under identical conditions (0 °C, 2 h) with a folded library of 43 RNAs containing systematically varied loop sizes and loop types.^12^ Loops were constructed of polyU and polyA sequence, to test extremes of base stacking and helicity. We employed deep sequencing to quantify and pinpoint sites of reaction by reverse transcriptase (RT) stops (Fig. 3a, S8 and S9†). Comparison of the reactivity of opposite enantiomers of a reagent provides diastereoselectivity data (Fig. 3b and S10†). The RT stop method involves single-hit conversions (*ca.* 0.1–1.0% of each reactive 2′-OH), so the data provide a measure of kinetic selectivity of acylation at early timepoints of reaction.

### Reactivity varies with structural context

Plots of the data for positional reactivity of the two pairs of chiral molecules are shown in Fig. 3. The data show widely variable reactivity across diverse folded RNA structures (Fig. 3a), with loops showing elevated reactivity in general and paired regions low reactivity, as has been observed previously for achiral reagents.^3^

Interestingly, nucleotides in small bulge loops (*e.g*., B4, B5) and internal loops (I21, I22, I32) are the most reactive contexts for the chiral reagents in general, and indeed are more reactive than nucleotides in single-stranded RNAs (*e.g.* ss-polyA and ss-mixed sequence RNA (“ss-combo”) (Fig. 3a)). Unlike previous achiral compounds,^12,39^ certain small loops (B2, B3) that were highly reactive for achiral reagents proved to be relatively unreactive for the current chiral reagents. Also noteworthy for the chiral compounds is that small uridine loops are generally more reactive than those composed of adenosine nucleotides (Fig. S9†), which is the opposite of what was seen for previous achiral aryl species.^12,39^

Finally, we measured overall (averaged) selectivity of each compound ((*R*,*S*)-1, (*R*,*S*)-2) for acylation of unpaired loop residues over paired ones (stems) in the folded RNA library. This ratio has been used as a marker to evaluate acylating reagents for structure mapping; a high loop/stem (L/S) reactivity ratio indicates elevated signal over background. The data (Fig. 3c) show that each enantiomer has distinct loop/stem ratios, with compounds (*R*,*S*)-1 showing higher L/S values than (*R*,*S*)-2. The data for (*R*,*S*)-1 are particularly remarkable, with a higher value for (*S*)-1 than its mirror image; indeed, the L/S value of 8.5 for (*S*)-1 is considerably higher than that of all structure mapping acyl reagents currently in use.^12,40^

### Large variations in positional diastereoselectivity

Next we plotted diastereoselectivity at each position in the folded RNA library (Fig. 3b and S8†). The data overall show that one enantiomer on average is more reactive than the other, as seen in individual RNA studies above, thus confirming chiral selectivity in general for reactions with naturally configured (d)-RNA. Notably, positional diastereoselectivity varies widely with RNA structure (Fig. 3f, g, S10 and S11†). Most loop positions exhibited greater than 2 : 1 diastereoselectivity, and remarkably, we observed near-complete (∼100%) diastereoselectivity at dozens of loop positions in the folded RNAs at the low reaction conversions of the experiment. Consistent with the high-conversion mass spectrometry data, the (*S*) enantiomer is generally favored for scaffold 1, while the (*R*) enantiomer of 2 is favored at most positions (Fig. 3e).

Positional diastereoselectivity in the folded RNAs varied both among distinct loop types and sizes, as well as along the different positions within a single loop, apparently reflecting distinct chiral structural contexts. For example, very small hairpin loops (H2, H3) exhibit relatively low diastereoselectivity in reactions with scaffold 1 (Fig. 3f), and a three-way junction with a 3-nucleotide loop (T3) displays the opposite stereochemical preference relative to most other loops. We also evaluated diastereoselectivity with three single-stranded RNAs in the library (ssRNA): polyU, polyA, and a mixed A,C,G,U sequence (ss-combo). There was very little reactivity with polyA, as seen previously for achiral reagents, presumably reflecting the high helicity of this sequence due to the strong stacking of adenines.^41^ As with folded sequences, scaffolds 1 and 2 showed opposite diastereoselectivity that was significant especially for single-stranded polyU (Fig. 3e and S10†). Overall, stereoselectivity is comparatively low for these relatively unconstrained RNAs, suggesting that the more rigid structural contexts in small loops enable greater discrimination between mirror-image reagents. The data also establish that sequence effects appear to be small for stereoselectivity as compared with folded structure, which has a significantly larger influence (Fig. S11†).

To further explore the role of reagent stereochemistry in local nucleotide reactivity across this RNA library, we examined nucleotide-by-nucleotide positional correlations of reactivity of enantiomers of 1 and 2 (Fig. S12†). Interestingly, enantiomers of 2 (the more stereoselective scaffold) show very little correlation with each other, while the enantiomers of 1 show a substantial positive correlation. This is also the case for prior relatively flat aryl reagents NAIN3 and 1M7, which also correlate well in their positional reactivity.^12^ The results suggest that compounds with greater asymmetry can better sense differences in local chiral environment around each 2′-OH.

Averaging diastereoselectivity of reagent scaffolds 1 and 2 over all loops (Fig. 3e) revealed remarkable diastereoselectivity in loops, but almost no asymmetric discrimination in duplexes (stems). Taken together, our RNA library data confirm that 2′-OH acylation by these chiral acylimidazole reagents occurs primarily at unpaired loop nucleotides, and that reactivity and diastereoselectivity, at least at low reaction conversions, vary greatly with local RNA loop structure. Small loops (especially bulge and internal loops) tend to show elevated reactivity and diastereoselectivity at discrete positions.

### Chiral structure mapping

**Fig. 4:**
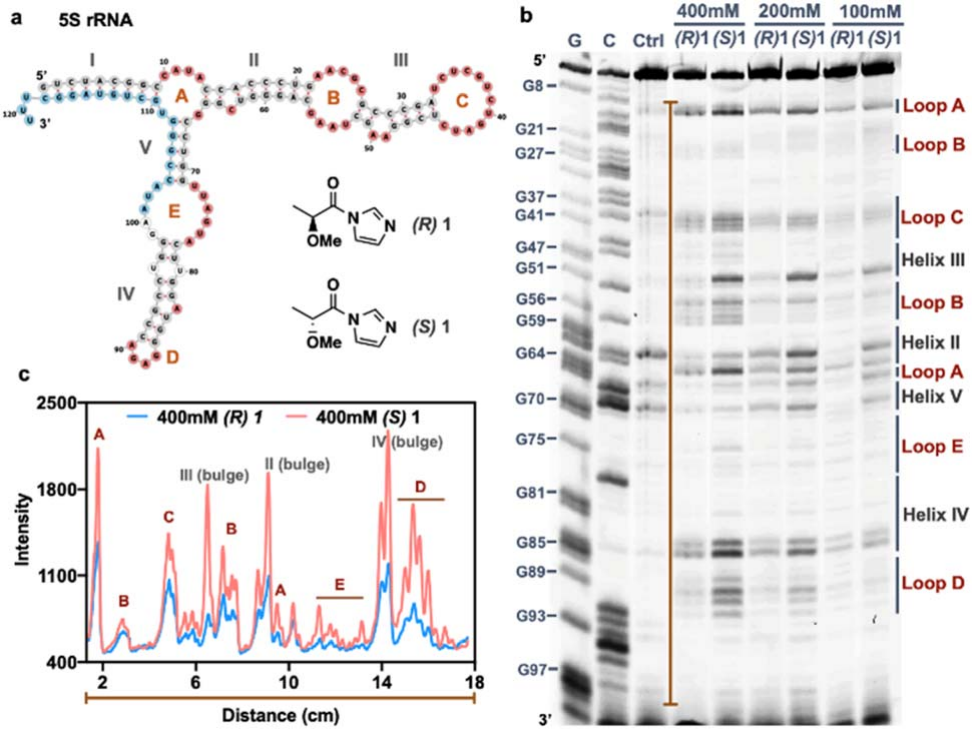
In-cell RNA structure mapping with chiral acylating reagents reveals elevated signal/background for a chiral reagent. (a) Secondary structure of human 5S rRNA generated by Vienna RNAfold with structure information from RNA central database. The helix regions (grey), detected loop regions (red: A–E and bulges in helices), and the primer binding site (light blue) for reverse transcriptase primer extension are shown. (b) In-cell SHAPE probing of human 5S rRNA with different concentration (100 mM, 200 mM, 400 mM) of enantiomers 1 ((*R*)-1 and (*S*)-1) for 10 min in HEK293 cells. The loop regions (red) and helix regions (grey) are denoted in the gel corresponding with the published 5S rRNA structure. Sequencing lanes are labeled with G and C for each nucleotide identification. (c) Plot profile of the band intensity along the orange line in (b) with the 400 mM condition, showing >2-fold higher peaks relative to baseline for (*S*) enantiomer (red line) over (*R*) enantiomer (blue line) in the detected loop regions.

Our structure-dependent sequencing data above revealed that chirality can have large effects in selectivity, as seen by extremely high loop/stem reactivity ratios for the (*S*) enantiomer of 1 (L/S = 8.5) as compared with the (*R*) variant (Fig. 3c). For comparison, established reagents 1M7 and NAIN3 have reported L/S reactivity ratios of 4.3 and 5.4, respectively;^12^ this difference suggests the possible future utility of chiral agents in RNA structure mapping with higher signal over background than is currently possible with the current generation of achiral mapping reagents. As a preliminary test of this possibility, we carried out SHAPE^3^ mapping studies of 5S ribosomal RNA in live HEK293 cells using the two enantiomers of 1. The data show that the reagent scaffold has intracellular reactivity similar to that of established compounds, achieving signal in 10 min (Fig. 4). Remarkably, the (*S*) enantiomer exhibits over twice the signal over background of the (*R*) enantiomer in mapping this intracellular RNA (Fig. 4b and c). Thus the early mapping data support the notion that chirality is an important consideration in design of RNA structure-mapping reagents.

### Applications in enantioselective conjugation and labeling

**Scheme 1:**
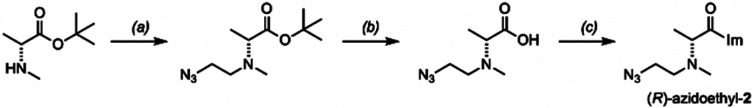
Synthesis of a chiral azide-functionalized analog of (*R*)-2 for local RNA labeling. Conditions: (a) azidoacetaldehyde, NaBH(OAc)_3_, DCM, 16 h, r.t. (73%); (c) HCl in 1,4-dioxane, DCM, 17 h, r.t. (87%); (d) CDI, DMSO, r.t., 2 h, quant. (as HCl salt). See ESI file† for synthesis of the (*S*) enantiomer and analytical data.

Our sequencing-based structure survey shows that diastereoselectivity of chiral acylating agents varies widely with position and structure in RNA. We next chose selected examples of small folded RNAs to investigate further, to evaluate whether the reactions could be carried out with diastereoselectivity on the scale of stoichiometric reactions, potentially enabling their use in localized conjugation and labeling. To test this possibility, we synthesized functionalizable variants of scaffold 2, replacing one methyl of the dimethylamino group with an azidoethyl group (Scheme 1); we confirmed that the syntheses did not appreciably racemize the alpha carbons (Fig. S13†). Experiments showed that this scaffold affords similar or slightly higher diastereoselectivity in RNA reaction as the parent scaffold 2 (Fig. S14 and S15†).

**Fig. 5:**
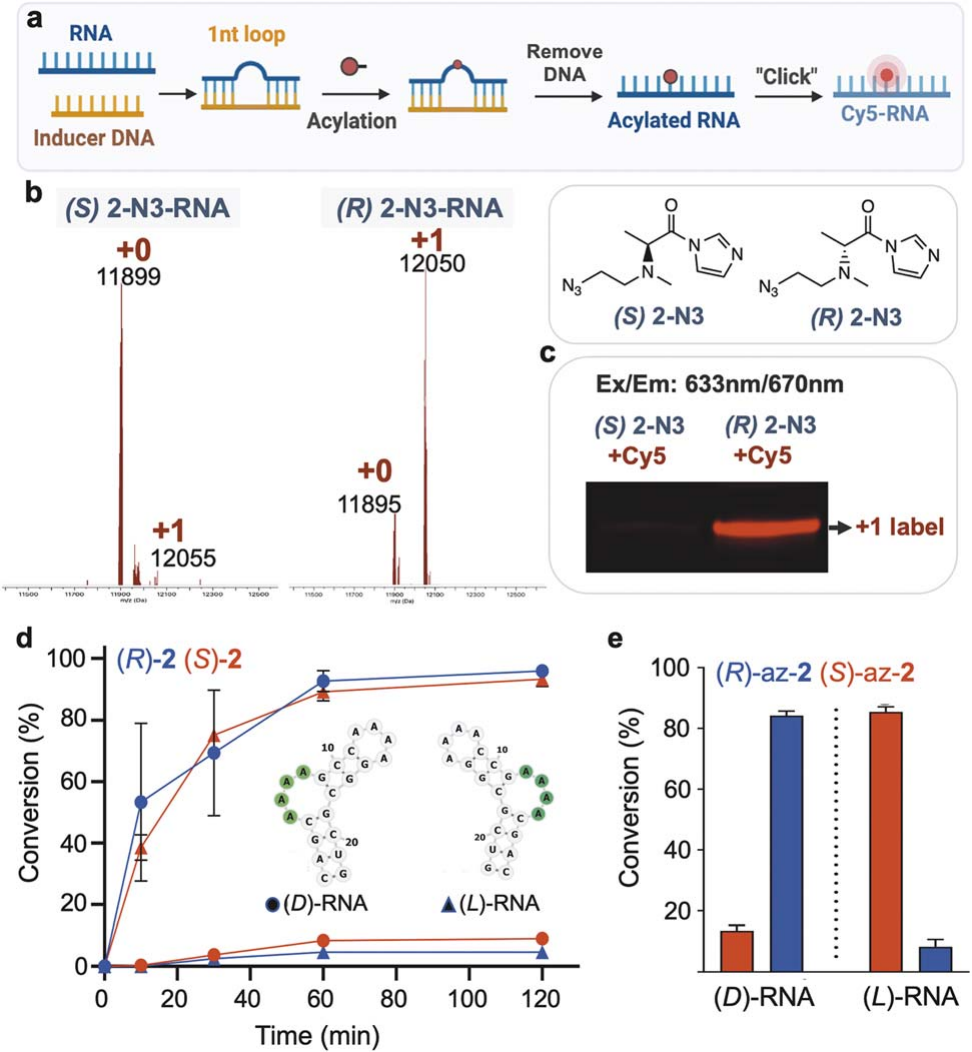
Stereoselective modification of small RNA loop motifs of natural and mirror-image RNAs. (a–c) Stereoselective and site-selective fluorescent labeling of native d-RNA by DNA-induced reaction of (*R*,*S*)-azidoethyl-2)-functionalized RNA with DBCO-Cy5 conjugate. (b) MALDI-TOF analysis of acylated RNA. The reaction was conducted with 6 mM (*R*,*S*)-2-N3 at 0 °C for 1 h and the labeling was analyzed by gel and visualized under red fluorescence channel (c). (d) Time course of reaction conversions of both enantiomers of a short folded RNA (B4A) using reagents (*R*,*S*)-2, (3 mM) showing stereoselectivity of (*R*)-2 for *D*-RNA and (*S*)-2 for l-RNA. Reactive bulge loop is marked in green. (e) Graph of RNA conversions at 2 h with chiral functionalizable reagents *N*-azidoethyl-2 (3 mM), showing mirror-image selective reactions of enantiomers with RNAs of opposite handedness. Data show average of 3 replicates ±s.d.

We then explored the possibility of acylation reactions of these chiral reagents with a bulged ribonucleotide in an RNA/DNA hybrid structure (Fig. 5a), to test the possibility of site-specific conjugation *via* induced loop formation.^4^ The results showed conversion yields at 1 h of 83% (*R*) and 5% (*S*) for the two enantiomers, yielding diastereoselectivity of >16 : 1 (Fig. 5b). We reacted the products with a commercial strained alkyne-functionalized Cy5 dye to prepare fluorescent-labeled adducts. Analysis by gel electrophoresis documents the much higher yield of the preferred enantiomer as seen by its strong labeling intensity (Fig. 5c), whereas the less-preferred enantiomer yields little visible fluorescence. This clearly demonstrates that chirality can have large influences in high-yield RNA labeling.

While most applications of modified RNAs involve strands of natural chirality (composed of d-ribose phosphodiester backbone), recent studies have investigated the properties and applications of enantiomeric (“mirror image” or “spiegelmer”) RNAs, with l-ribose as the source of chirality.^42–47^ Given the favorable properties of *enantio*-RNAs in applications such as aptameric ligands in biological settings, we investigated a further test of chiral selectivity in conjugation and labeling of enantiomeric RNA. We carried out reactions of d- and l-enantiomers of a short RNA having a reactive bulge loop structure (sequence B4A from the above library) using each enantiomer of the dimethylalanine reagent 2. MS data (Fig. 5d) documented conversions at 1 h of ∼90% for each “matched” pair of reagents with RNA ((*R*) with d-RNA; (*S*) with l-RNA), and considerably lower 5–8% conversions for the “mismatched” pairs. Diastereoselectivity was over 100 : 1 at early time points and averaged 24 : 1 at ∼70% conversion. Thus, highly enantioselective reaction was seen for both native and mirror-image RNAs using reagents of appropriately matched handedness. Extending this to a functionalizable reagent, we reacted the enantiomers of azidoethyl-2 with the d- and l-RNAs, resulting in preparative conversions of 85% and diastereoselectivities averaging 8 : 1 (Fig. 5e). The experiments confirm that both antipodes of the synthesized azidoethyl-alanine acylimidazole reagent do indeed function normally in acylation, allowing us to rule out degradation or impurity in one enantiomer leading to misinterpretation of selectivity. Taken together, the new findings enable selective conjugation of natural RNA or its mirror image, which was previously not possible.

### Discussion and conclusions

Our experiments establish that chiral acylimidazole reagents with appropriate asymmetric substitution can exhibit substantial levels of diastereoselectivity in RNA reaction. This is, to our knowledge, the first report of chiral selectivity for acylation of non-homopolymer RNAs. Multiple surprising findings arise from the study regarding unusual reactivity and selectivity (*vide infra*). Our results add support to early reports of amino acid reactions with dinucleotides,^33–35^ confirming that amino acid derivatives can indeed react at 2′-OH. The current work identifies new reagents and RNA folded structures that exhibit considerably higher yields and greater diastereoselectivity than previously seen, establishes new reagents that enable high-yielding and highly selective RNA modification, and demonstrates new applications of chirality.

It is clear from our initial screening data that reagent structure can have large effects on selectivity, as most of the reagents tested here show modest stereoselectivity. However, for selected scaffolds, we observe near-complete diastereoselectivity at low conversions at specific sites in folded RNA, and up to 24 : 1 diastereoselectivity in preparative reactions. These findings add a new physicochemical dimension to the study of RNA recognition and reaction. Our findings confirm chirality as a significant and overlooked structural mechanism for selectivity control in covalent RNA recognition and conjugation.

Our new results add the dimension of asymmetry as an important influencing factor in reagent design, going beyond the effects of alkyl/aryl substitution and steric bulk.^12,39,40^ For example, our experiments have identified a chiral acylating species, (*S*)-1, that exhibits exceptionally high loop/stem selectivity relative to its enantiomer, and indeed considerably higher than that of any reported agent to date. No previous chiral compound has been employed in mapping of folded RNA structure. In our preliminary tests, this resulted in markedly improved signal over background in intracellular RNA mapping. Further exploration of this finding seems warranted, with the aim of developing future high-performing generations of transcriptome-probing reagents.

In addition, given the rapid development of RNAs as therapeutic agents, establishing methods to locally substitute RNA for conjugation is also of substantial interest;^5,45,48^ however, it is significantly challenging, due to the large number of chemically similar groups in the biopolymer. In this light, our observation of the highly selective stoichiometric labeling reaction of (*R*)-azidoethyl-2 seems promising (Fig. 4b and e). Taken together, the current studies establish the use of reagent chirality coupled with local RNA macro-chirality as a potentially promising strategy for enhancing local selectivity in RNA reaction; more work will be needed to establish ideal sequences and to test applications in longer biologically relevant RNAs.

It is interesting and noteworthy that α-amino compounds in this study (*e.g.*2, 8) exhibit the opposite diastereoselectivity for RNA, for the most part, as the structurally analogous α-alkoxy compounds (*e.g.*1, 10). This suggests that the two classes of compounds adopt distinct conformations that favor approach of 2′-OH to the opposite faces of the reactive carbonyls. As a possible explanation (Fig. S16†), we hypothesize that steric effects of the larger dialkylamino group relative to the alkoxy group play a role in this difference. More studies will be needed to shed light on this divergence, and a better understanding could possibly lead to future reagent designs with altered or improved selectivity.

Also remarkable and unexpected is our finding of exceptionally high reactivity by compounds (*R*,*S*)-2, and the (*R*) enantiomer in particular, in acylation of RNA. Indeed, (*R*)-2 is the most highly RNA-reactive compound yet described in the literature to our knowledge. An experiment benchmarking the reactivity of enantiomers of 2 in comparison to the most commonly applied previous acylation scaffolds employed in RNA research (1M7 and NAIN3) underscores this marked difference (Fig. S17†). It seems likely that relative to non-basic alkoxy scaffolds, the protonation of the amine group in these *N*,*N*-dimethylaminoalanine derivatives at the pH of our reactions plays a role, by inductively activating the carbonyl carbon to greater electrophilicity, as supported by the high reactivity of 2 relative to non-protonated compound 1. Similarly, proline derivative 8 also shows higher reactivity (by a factor of >50-fold) than structurally related tetrahydrofuran scaffold 10. Comparisons of reactivity of (*S*)-2 and dimethylglycine (DMG) acylimidazoles (which differ by only a methyl group) reveal similar RNA reactivity and half-lives for hydrolysis (Fig. S3†), indicating that the methyl substitution in (*S*) has little effect on carbonyl reactivity with water in this isomer. However, the (*R*) enantiomer of 2 shows higher reactivity for RNA than the (*S*) isomer (by a factor of roughly ten), and acylates the biopolymer with >90% conversions even at the remarkably low concentration of 100 μM, one thousand-fold lower than common concentrations of RNA acylating agents. This suggests that the methyl group substitution in the (*R*) configuration confers specially elevated reactivity with RNA.

In an effort to better understand the high reactivity of (*R*)-2, we considered whether the compound might bind RNA noncovalently. We followed up by performing a titration of concentration *vs.* conversion, and by testing the effects of competition by a chiral nonreactive analogue of (*R*)-2 (Fig. S18 and S19†). The competitor did modestly reduce RNA yields at high concentrations, suggestive of binding, but had similar effects on the less reactive achiral compound DMG as well (Fig. S19†). Given the similar reactivity of (*R*)-2 in multiple loop types (Fig. 3b), any RNA binding by this compound appears to be nonselective. More work is needed to fully understand the origin of this favorable chiral methyl effect, although the special effects of methyl substitution conferring potency have been noted widely for protein-targeted drugs.^49^

Taken together, these data establish new ways to achieve selectivity in RNA conjugation reactions, by taking advantage of stereoselective recognition between asymmetric small-molecule reagents and the local chiral environment of RNA 2′-OH groups. The chiral reagent scaffolds studied here are in several cases commercially available as carboxylic acids and are easily activated to acylimidazole form in dry DMSO in one step without purification to yield stock solutions. Thus our findings, and extensions to new chiral molecular scaffolds, should be readily accessible to other researchers. We envision multiple outcomes that may result in the long term. For example, it may prove possible to develop chiral probes that are specific for certain folded RNA motifs. Second, further study may lead to the identification of folded structural contexts that may be functionalized selectively over more common motifs (such as duplex regions) in RNAs, enabling improved site selectivity in localized conjugation and labeling of larger RNAs. Future work will be required to explore these possibilities.

## Data availability

The datasets supporting this article have been uploaded as part of the ESI.† The sequencing data have been deposited at the Gene Expression Omnibus (GEO) database repository, and can be obtained by searching the article title.

## Author contributions

R. S. synthesized all compounds, performed screening and optimization of acylating agent reactions with RNA, and determined the stereoselectivity of all compounds. L. X. performed experiments to probe chiral selectivity on folded RNA, performed in-cell RNA structure mapping, and performed site-selective RNA modification experiments. S. C. synthesized, purified, and characterized L-RNA oligonucleotides. E. T. K. designed the project and supervised experiments. R. S., L. X., and E. T. K. wrote the paper with input from all authors.

## Conflicts of interest

There are no conflicts of interest to declare.

## Supplementary Material

SC-014-D3SC03067A-s001
