# Supplementary material for: Stereoselective RNA reaction with chiral 2′-OH acylating agents
Source: Chem Sci. 2023 Nov 7;14(45):13235–43. doi: 10.1039/d3sc03067a (PMC10664579; doi:10.1039/d3sc03067a)
Supplement: SC-014-D3SC03067A-s001 [file SC-014-D3SC03067A-s001.pdf]

Electronic Supplementary Material (ESI) for Chemical Science.
This journal is © The Royal Society of Chemistry 2023

Supporting Information

Stereoselective RNA Reaction with Chiral 2′-OH Acylating Agents

Ryuta Shioi[#], Lu Xiao[#], Sayantan Chatterjee, Eric T. Kool[*]

Department of Chemistry, Stanford University, Stanford, CA 94305
[#]Equal contributions.
*Author to whom correspondence should be addressed: kool@stanford.edu

Table of contents

Materials and instrumentation S2
Supplementary Figures S8
Synthetic procedures S27
References S41

Materials and instrumentation

Instrumentation

NMR Spectroscopy: All NMR spectra were recorded at the Stanford University Department of Chemistry NMR facility. Varian 300 MHz, 400 MHz and 500 MHz NMR instruments were used to record the 1H and 13C spectra. The spectra were analyzed using MNova software.

ESI-MS: All ESI-MS spectra were recorded at the Stanford University Mass Spectrometry Facility. The instrument used was a Waters 2795 HPLC system with dual wavelength UV detector, and ZQ single quadrupole MS with electrospray ionization source

MALDI-TOF MS: All MALDI-TOF spectra were recorded at the Stanford University Mass Spectrometry facility, using the Bruker Daltonik Microflex MALDI-TOF spectrometer equipped with an N_2 laser. All spectra were recorded in linear negative mode and samples were plated on an MSP Anchorchip 96 target plate. 0.3 M trihydroxyacetophenone in EtOH (matrix) and 0.1 M aqueous ammonium citrate (co-matrix) were mixed in a 2:1 ratio by volume to be used as a matrix mix for MALDI. This mix was always freshly prepared before analysis. After RNA precipitation, the RNA pellet was redissolved in RNAse-free water to prepare a 10 µM sample solution. 1 µL of this solution was transferred to the target plate and dried under an Ar stream. 1 µL of the matrix mix was then added directly on top of the dried sample and completely dried under Ar stream. The spectral data was then recorded using Flex Control software (Bruker), and analyzed using MNova (Mestrenova).

Chemical reagents

Reagents were purchased from Sigma-Aldrich unless specified otherwise. (*R*)/(*S*) 2-Methoxypropanoic acid, (*R*) /(*S*) *N*,*N*-Dimethylalanine and D/L Alanine and (*R*)/(*S*) Methoxyphenylacetic acid, (*R*)/(*S*) 2-(*tert*-Butoxycarbonylamino) propanoic acid and (*R*)/(*S*) 2-(Benzyloxycarbonyllamino) propanoic acid, (*R*)/(*S*) α-Methyl-3-pyridinemethanol and (*R*)/(*S*) 1-Methylpyrrolidine-2-carboxylic acid, (*R*))/(*S*) Methyl Pyrrolidine-3-carboxylate hydrochloride and (*R*)/(*S*) Tetrahydro-2-furancarboxylic acid, Methyl (*R*) /(*S*) 2,2-dimethyl-1,3-dioxolane-4-carboxylate and N-Methyl-D/L-alanine 1,1-dimethylethyl ester were purchased from Ambeed. 1H-Imidazole-1-sulfonyl azide hydrochloride was purchased from Biosynth Product. Azidoethanol: Acrotein was purchased from ChemBio. Dess-Martin periodinaneNaBH(OAc)$_3$ was purchased from A2B Chem.

Biological reagents

RiboLock RNase inhibitor, FastAP thermosensitive alkaline phosphatase, SuperScript™ III reverse transcriptase kit, RNase cocktail enzyme mix, RNase H were purchased from ThermoFisher Scientific™. CircLigase II ssDNA ligase was purchased from Epicentre. RQ1 RNase-Free DNase I was purchased from Promega. T4 polynucleotide kinase, T4 RNA ligase 1, Phusion high-fidelity (HF) PCR master mix and Q5 hot start high fidelity PCR master mix were ordered from New England Biolabs. RNA clean-up and concentrator-5 column and DNA clean-up and concentrator-5 column were purchased from Zymo Research. Corning Costar Spin-X centrifuge filters, 0.45 μm and Amicon Ultra-0.5 3K/10K-Centrifugal Filter Unit were purchased from Millipore-Sigma. MiniElute gel extraction Kit was purchased from QIAGEN. All DNA sequences were purchased from IDT.

General RNA acylation procedure for MALDI-TOF analysis

RNA Reactions with Chiral Acylating Reagents: In a sterile 200 μL PCR tube, 3.3 μL of 3.3X buffer (333 mM MOPS pH 7.5, 333 mM NaCl, 20 mM $MgCl_2$ in water), was mixed with 4.7 μL of 21.3 μM RNA stock solution. A fresh stock of chiral acylating reagent was prepared in DMSO (5x final concentration); 2 μL of stock solution was added to the RNA reaction. This reaction was incubated for 2 hr at 0 °C and the RNA was subsequently purified by ethanol precipitation. The level of RNA modification was measured by MALDI-TOF M/S and analyzed using MestReNova™ software.

Ethanol precipitation of RNA reactions: For an RNA reaction with total volume 10 μL, 90 μL RNA precipitation solution (0.33 M NaOAc (pH 5.2) in water containing 0.2 mg/mL glycogen) was added and mixed well. 300 μL of ice-cold absolute ethanol was then added, and the mixture mixed by vortexing for at least 30 s. After storage at -80 °C overnight, the mixture was centrifuged at 14.8k RPM for 60 mins at 4 °C. The supernatant was discarded to obtain a pellet, which was washed with 70% ethanol. The obtained pellet was air dried for 15 min and subsequently either stored at -80 °C for future use or dissolved in water/PBS for direct use in further experiments.

Reactivity analysis by sequencing

We employed the published method[1] to react different reagents with RNA libraries under optimized conditions. Briefly, each RNA in the library was first folded individually and then equally mixed with others in the set, assembling separate structured A-loop and U-loop RNA libraries. In a final 10 μL acylation reaction, 2 μL 25 μM structured library RNA mixtures were added with 2 μL 5x acylating reagents ((*R, S*)-**1** and (*R, S*)-**2**) in DMSO (+) or DMSO (−) as mock treatment and 3.3 μL 3.3×SHAPE buffer (800 mM MOPS, pH 7.5, 48 mM $MgCl_2$, 800 mM NaCl). The reaction was incubated at 0 °C for the 2h at 50mM for **1** and 100μM for **2** and was quenched by DTT with 1.2

equivalent amount of acylating reagents at 0 °C for 15 min. Acylated RNA libraries were purified by 3K-Amicon centrifuge filter, washing with RNase-free water. The purified and reacted RNAs solutions were aliquoted to ~500ng per tube and were freeze-dried on a lyophilizer prior to deep-sequencing library preparation.

To prepare the library for deep sequencing, we followed the method previously reported[1], including RNA 3'-end repair and ligation, RNA size selection, reverse transcription with barcode-encoded primers for each reagent reacted library, cDNA purification and circularization, library amplification, and library size selection. For detailed experimental procedures see ref[1]. Samples reacting with different reagents were pooled together for multiplexing to proceed with sequencing.

Paired-end sequencing was performed on an Illumina HiSeq sequencer, yielding approximately 120 million raw reads. We utilized our previous method[1] to analyze our sequencing data, which built on the established icSHAPE pipeline[2] with some modifications in parameter setting. Briefly, the raw sequencing data was processed by demultiplexing according to the barcode, collapsing to remove PCR duplicates, primer and linker trimming. RNA library index files were built, and trimmed reads were mapped to sequences in each RNA library. The absolute RT-stop frequencies for each condition were calculated following the scripts in the pipeline. We defined the positional SHAPE reactivity of each nucleotide (R) as the subtraction of background reverse transcription stops (mock DMSO libraries) adjusted by the background base density from reverse transcription stops of the acylated libraries adjusted by the sample base density: $R = (\text{RT-Stop}_{\text{sample}} / \text{base\_density}_{\text{sample}}) - (\text{RT-Stop}_{\text{DMSO}} / \text{base\_density}_{\text{DMSO}})$.

In-cell RNA SHAPE probing. HEK293 cells in a 15-cm plate were washed with 10 mL of PBS once, then scraped off with 4 mL of 1xPBS into a 15ml falcon tube. Cells were centrifuged at 1000g for 2 min at 25℃ and the supernatant was removed. The cell pellets were resuspended in 450 µL 1xPBS and transferred to a 1.5mL Eppendorf tube. 50 µL of 10x stock chiral compound in DMSO (4 M, 2 M, 1 M (*R,S*)-**1**) was added to the cells and incubated at 25℃ with rotation for 10min. The reactions were stopped by proceeding with total RNA isolation. Cells were lysed with 6 mL of Trizol LS by vortexing. 1.2 mL of chloroform was then added. The resulting mixture was vortexed and incubated at room temperature for 5 min, followed by centrifugation at 2500g for 15 min at 4℃. The aqueous phase was then mixed with 1× volume of 96% ethanol and purified using a Quick-RNA MidiPrep kit following the manufacturer's instructions.

PAGE analysis of reverse transcriptase (RT) stops. 5 µg isolated total RNA was mixed with 2 pmol Cy5-labeled 5S rRNA RT primer and 0.5 µL 10 mM dNTP mix (for sequencing lane, ddNTP:dNTP=8:1), and incubated at 95℃ for 2 min; then samples were cooled to 4℃ by stepping down 2℃/s. 2 µL 5x First-Strand Buffer, 1 µL 0.1 M DTT, 0.25 µL RiboLock and 0.25 µL

SuperScript III (200 U/μl) were added to the final volume of 10 μL. The reaction was incubated with the following program: 25℃ for 10 min, 52℃ for 50 min, and 55℃ for 50 min. After the reverse transcription reaction, 1 μL 1M NaOH was added and incubated at 95℃ for 3 min. Then 11 μL loading dye (8 M Urea, 0.05% Orange G, 0.05% Bromophenol blue) was added, and the mixture was denatured at 95℃ for 3 min and loaded on a denaturing 8% PAGE gel. Products were separated in the gel with 1x TBE running buffer (pH 8.3), 25mA, ~2.5 h. The cDNA gel was visualized by fluorescence imaging.

Stereoselective fluorescent labeling of native *d*-RNA.

50 pmol of RNA and 80 pmol of corresponding helper DNA were heated in folding buffer containing 50 mM NaCl to 95℃ for 3 min and chilled on ice to form an RNA-DNA duplex with 1nt U-bulge. To the annealed solutions were added 3.3 μL 3.3xSHAPE buffer and 2 μL 30mM (*R,S*)-azidoethyl-**2** in dry DMSO. The mixture was incubated at 0℃ for 1h and quenched by equivalent amout of DTT. 3 μL 1U/ μL DNase I was added to digest the helper DNAs at 37℃ for 1h. After reaction, the RNA was purified by Zymo RNA C5 concentrator column by adding 40 μL H_2O, 50 μL RNA binding buffer and 200 μL ethanol for small RNA purification procedure. 5 μM azido-functionalized RNA was further reacted with 50 μM Cy5-DBCO in a total 10 μL reaction in 1xPBS buffer at 37℃ for 3h. After labeling reaction, the RNA was precipitated by adding 90 μL of precipitation buffer (0.33M NaOAc, pH 5.2, glycogen 0.2 mg/mL) for gel analysis and fluorescence imaging.

Table S1. Oligonucleotides used in this work

**Name**	**Sequence (left to right: 5′ to 3′)**
**RNA oligo**	
ss RNA	ACAAUUAUCCUAUGAGCGGU
BL RNA	AGACAGCCUUUUUGGCGUCU
HP RNA	ACUGACUGAAACAGUCAGU
B4A	CAGCAAAAGCCAAAAGGCGCUG
I32A	CACCAAAGCCAAAAGGCAAGGUG
**DNA oligo**	
ss DNA	ACAATTATCCTATGAGCGGT

Name	Sequence (left to right: 5' to 3')
**A-loop RNA library**	
H2A	CGCGAACGCG
H3A	CCAGAAACUGG
H4A	UCCGAAAACGGA
H5A	ACUGAAAAACAGU
H6A	GGUGAAAAAACACC
H7A	AGGGAAAAAAACCCU
B1A	UAACAGCCAAAAGGCGUUA
B2A	AGACAAGCCAAAAGGCGUCU
B3A	GUCCAAAGCCAAAAGGCGGAC
B4A	CAGCAAAAGCCAAAAGGCGCUG
B5A	CCUCAAAAAGCCAAAAGGCGAGG
I11A	AUUCAGCCAAAAGGCAGAAU
I21A	GAUCAAGCCAAAAGGCAGAUC
I22A	CUACAAGCCAAAAGGCAAGUAG
I32A	CACCAAAGCCAAAAGGCAAGGUG
I33A	GUGCAAAGCCAAAAGGCAAAGCAC
T0A	CGUCAGGGCAAAAGCCCCGAAAACGGCUGACG
T1A	GCACAGGGCAAAAGCCCCGAAAACGGACUGUGC
T2A	UCGCAGGGCAAAAGCCCCGAAAACGGAACUGCGA
T3A	AGCCAGGGCAAAAGCCCCGAAAACGGAAACUGGCU
ss-polyA	AAAAAAAAAAAAAAAAAAAA
ss-combo	ACAAUUAUCCUAUGAGCGGU

**U-loop RNA library**	
H2U	CGCGUUCGCG
H3U	CCAGUUUCUGG
H4U	UCCGUUUUCGGA
H5U	ACUGUUUUUCAGU
H6U	GGUGUUUUUUCACC
H7U	AGGCUUUUUUUGCCU
B1U	UAAGUGCCUUUUGGCCUUA
B2U	AGAGUUGCCUUUUGGCCUCU
B3U	GUCGUUUGCCUUUUGGCCGAC
B4U	CAGGUUUUGCCUUUUGGCCCUG
B5U	CCUGUUUUUGCCUUUUGGCCAGG
I11U	AUUCUGCCUUUUGGCUGAAU
I21U	GAUCUUGCCUUUUGGCUGAUC
I22U	CUACUUGCCUUUUGGCUUGUAG
I32U	CACCUUUGCCUUUUGGCUUGGUG
I33U	GUGCUUUGCCUUUUGGCUUUGCAC
T0U	CGUCAGGGCUUUUGCCCCGUUUUCGGCUGACG
T1U	GCACAGGGCUUUUGCCCCGUUUUCGGUCUGUGC
T2U	UCGCAGGGCUUUUGCCCCGUUUUCGGUUCUGCGA
T3U	AGCCAGGGCUUUUGCCCCGUUUUCGGUUUCUGGCU
ss-polyU	UUUUUUUUUUUUUUUUUUUU
ss-combo	ACAAUUAUCCUAUGAGCGGU

Supplementary Figures

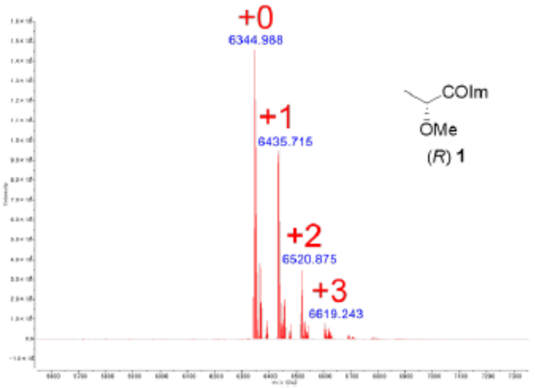

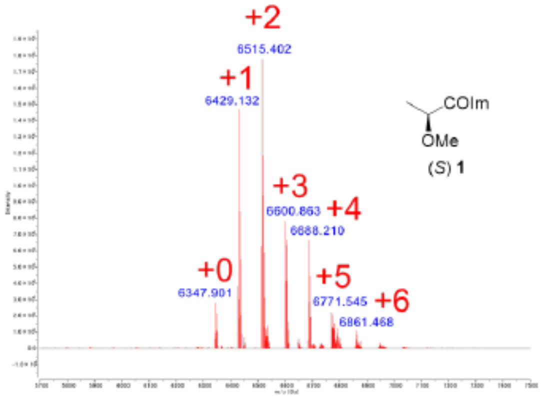

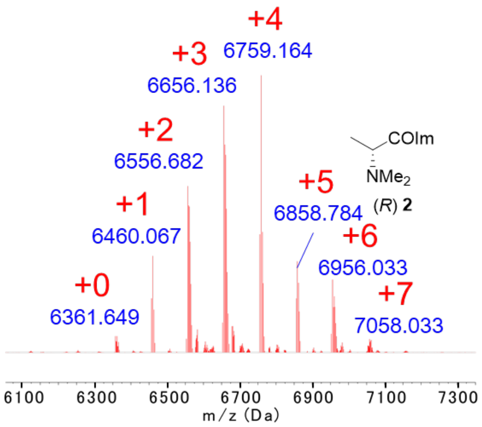

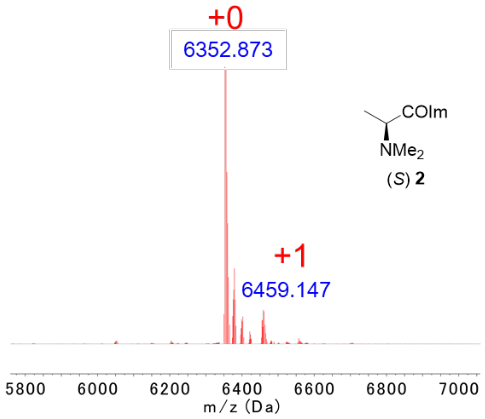

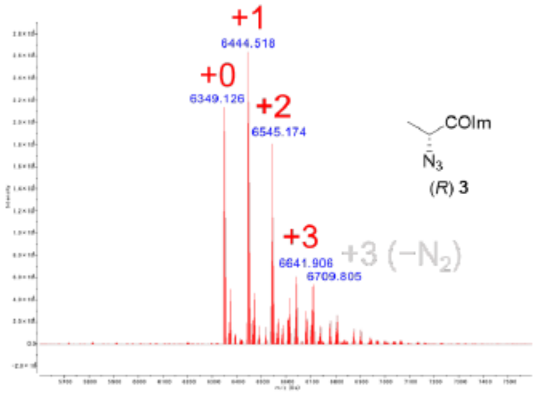
+1
6444.518
+0
6349.126
+2
6545.174
+3
6641.906
+3 (−N2)
6709.805
COIm
N3
(R) 3

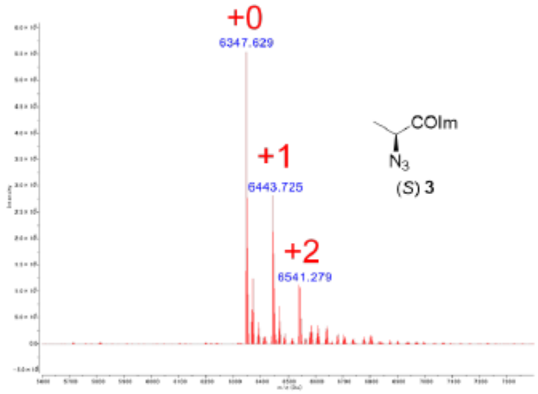
+0
6347.629
+1
6443.725
+2
6541.279
COIm
N3
(S) 3

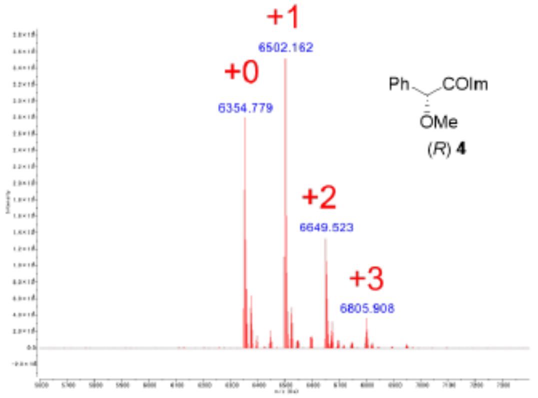
+1
6502.162
+0
6354.779
+2
6649.523
+3
6805.908
Ph
COIm
OMe
(R) 4

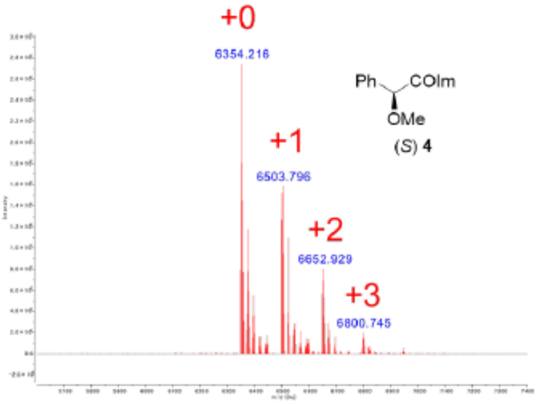
+0
6354.216
+1
6503.796
+2
6652.929
+3
6800.745
Ph
COIm
OMe
(S) 4

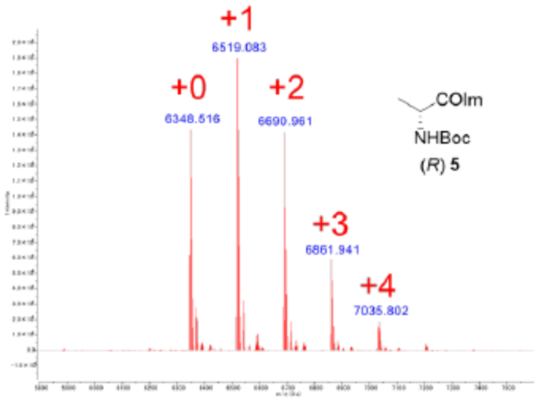
+1
6519.083
+0
6348.516
+2
6690.961
+3
6861.941
+4
7035.802
COIm
NHBoc
(R) 5

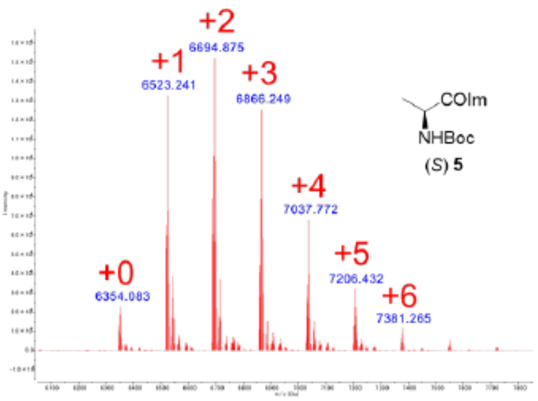
+2
6694.875
+1
6523.241
+3
6866.249
+4
7037.772
+5
7206.432
+6
7381.265
+0
6354.083
COIm
NHBoc
(S) 5

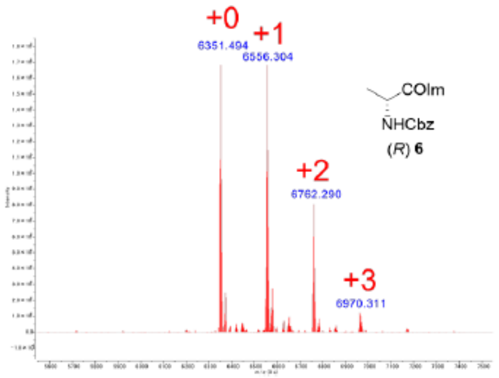
+0
6351.494
+1
6556.304
+2
6762.290
+3
6970.311
COIm
NHCbz
(R) 6

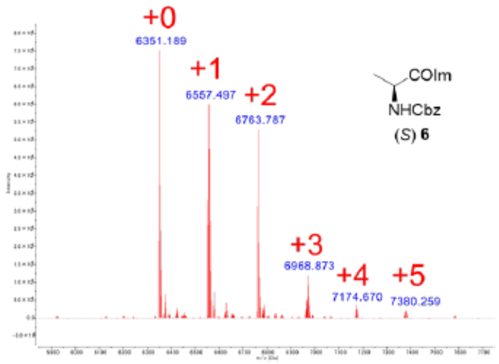
+0
6351.189
+1
6557.497
+2
6763.787
+3
6968.873
+4
7174.670
+5
7380.259
COIm
NHCbz
(S) 6

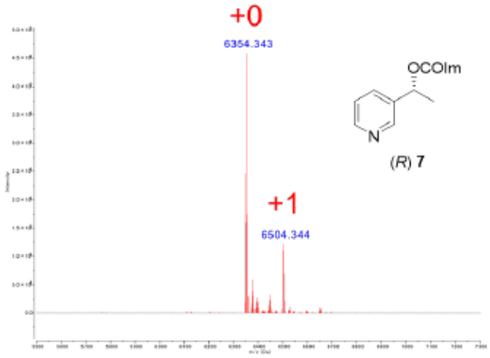
+0
6354.343
+1
6504.344
OCOIm
N
(R) 7

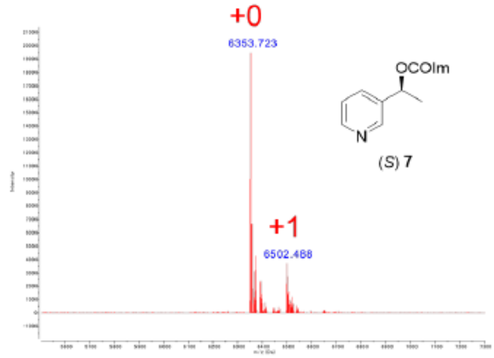
+0
6353.723
+1
6502.488
OCOIm
N
(S) 7

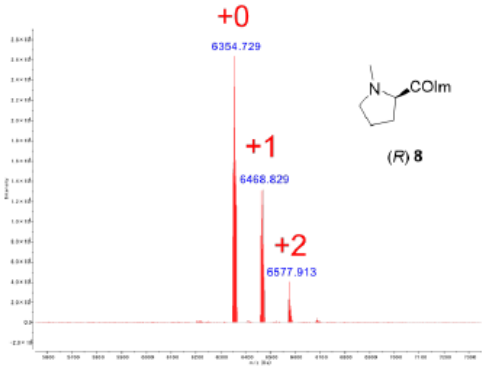
+0
6354.729
+1
6468.829
+2
6577.913
N
COIm
(R) 8

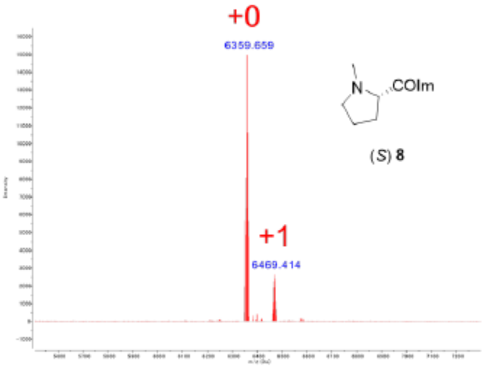
+0
6359.659
+1
6469.414
N
COIm
(S) 8

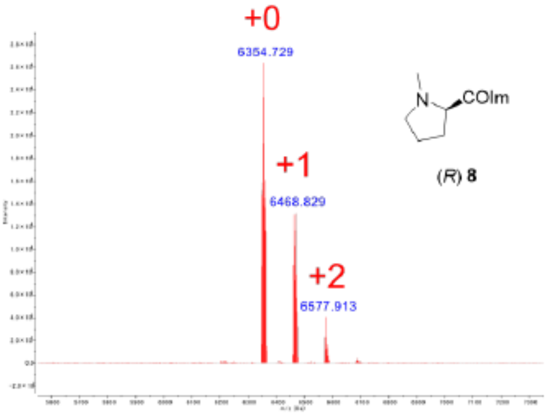
+0
6354.729
+1
6468.829
+2
6577.913
COIm
(R) 8

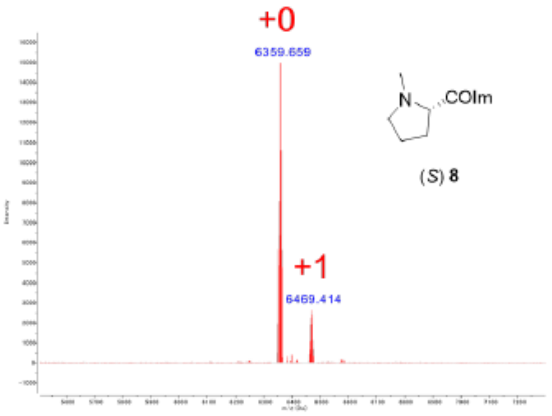
+0
6359.659
+1
6469.414
COIm
(S) 8

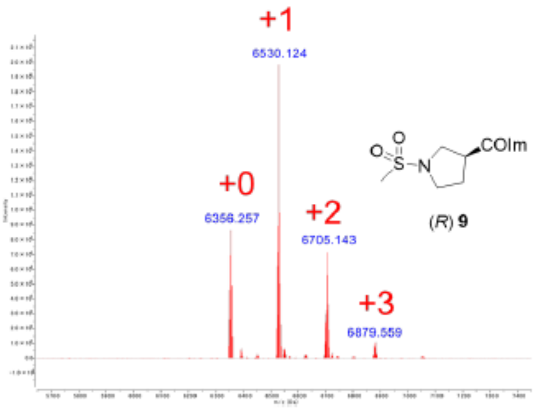
+1
6530.124
+0
6356.257
+2
6705.143
+3
6879.559
COIm
(R) 9

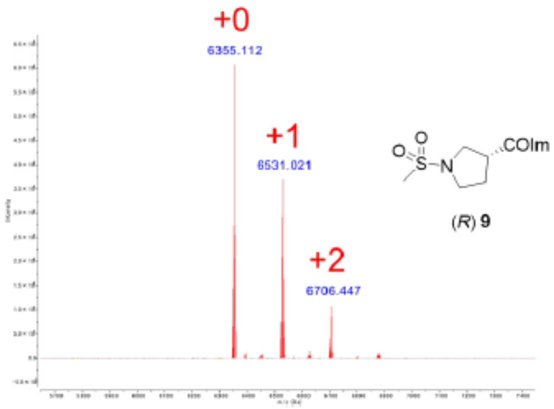
+0
6355.112
+1
6531.021
+2
6706.447
COIm
(R) 9

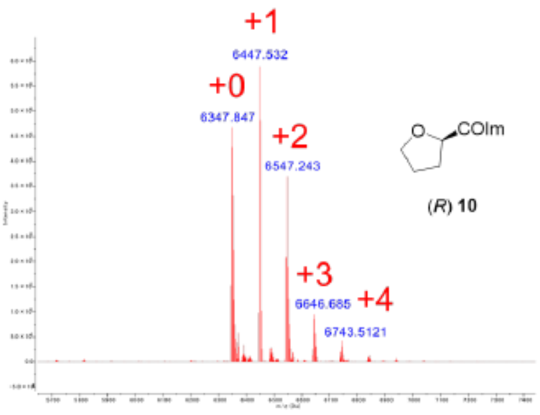
+1
6447.532
+0
6347.847
+2
6547.243
+3
6646.685
+4
6743.5121
COIm
(R) 10

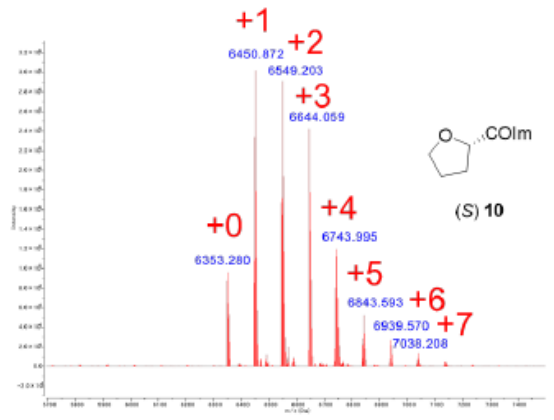
+1
6450.872
+2
6549.203
+3
6644.059
+0
6353.280
+4
6743.995
+5
6843.593
+6
6939.570
+7
7038.208
COIm
(S) 10

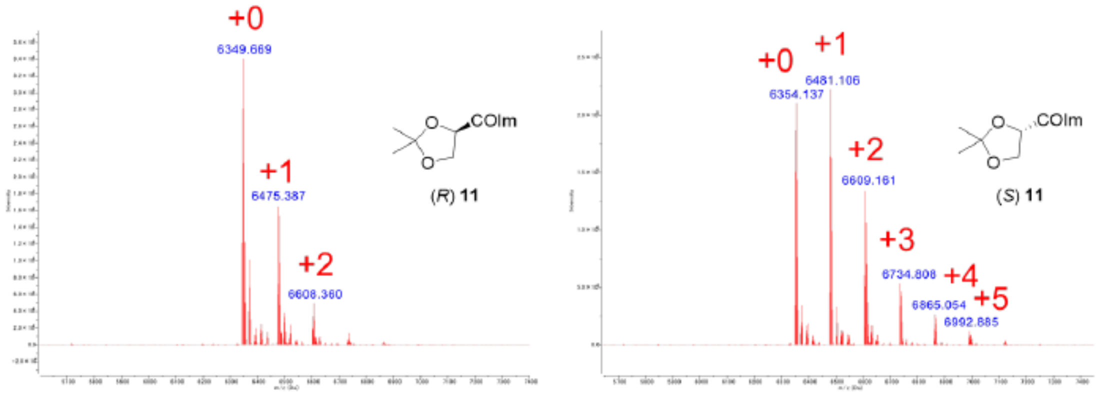

Figure S1. Representative MALDI-TOF mass spectrometry acylation data for reaction of 18nt single-stranded tRNA fragment with chiral acylimidazole reagents. Red numerals indicate number of adducts per RNA strand. Conditions are given in Table 1 (main text).

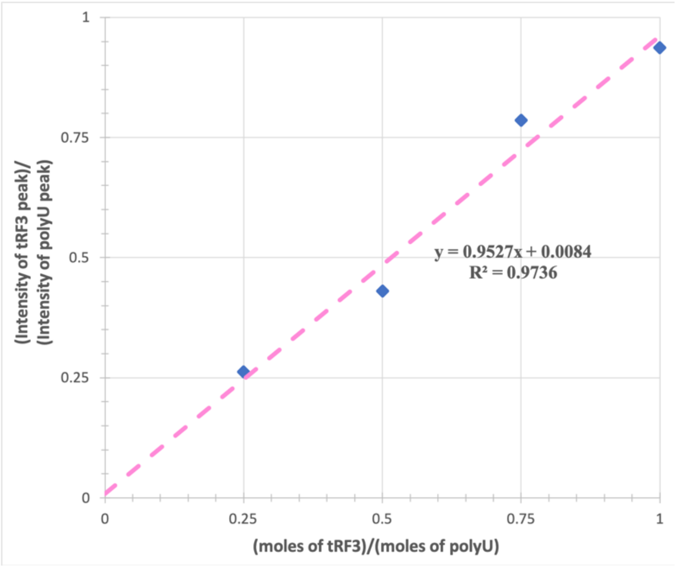

Figure S2. Quantitative use of MALDI-TOF MS for determining yields in small RNAs shows good linearity. Aqueous solutions of 20 μM U_{20} RNA (internal standard) were prepared and four such identical solutions were spiked with tRF3 RNA to final concentrations of 5 μM, 10 μM, 15μM and 20 μM. MALDI-TOF spectra were recorded following the procedure described above. The ratio of MALDI peak intensities of tRF3 and U_{20} RNAs was found to be linearly related to the mole ratio of tRF3 and U_{20} RNA. Data are from Chatterjee et al.[3]

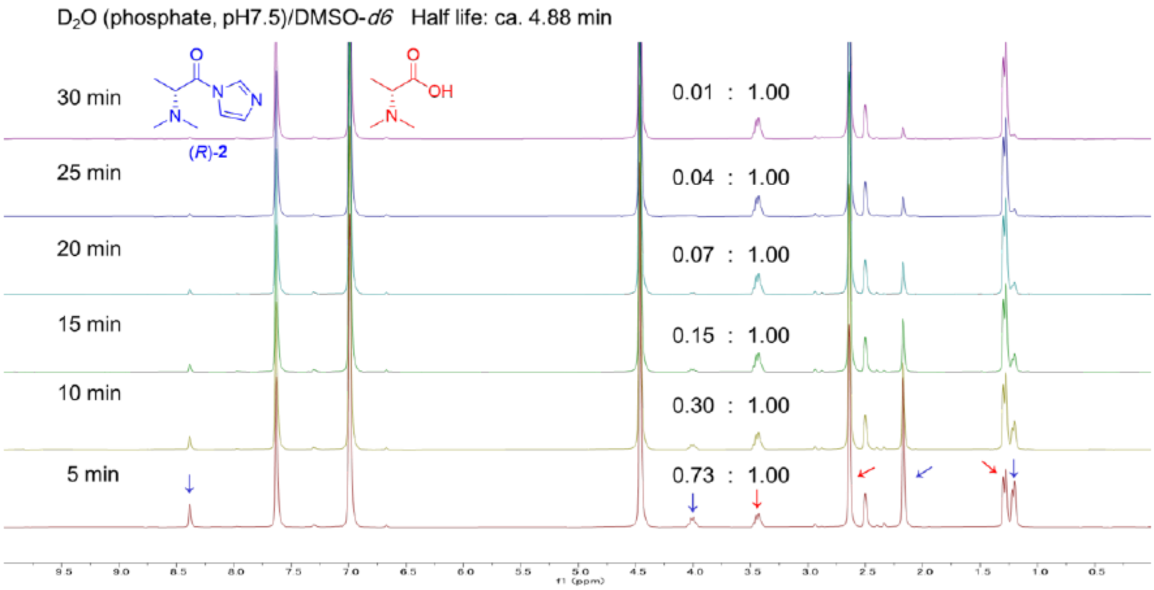
D$_2$O (phosphate, pH7.5)/DMSO-*d6* Half life: ca. 4.88 min
(*R*)-2
30 min 0.01 : 1.00
25 min 0.04 : 1.00
20 min 0.07 : 1.00
15 min 0.15 : 1.00
10 min 0.30 : 1.00
5 min 0.73 : 1.00
f1 (ppm)

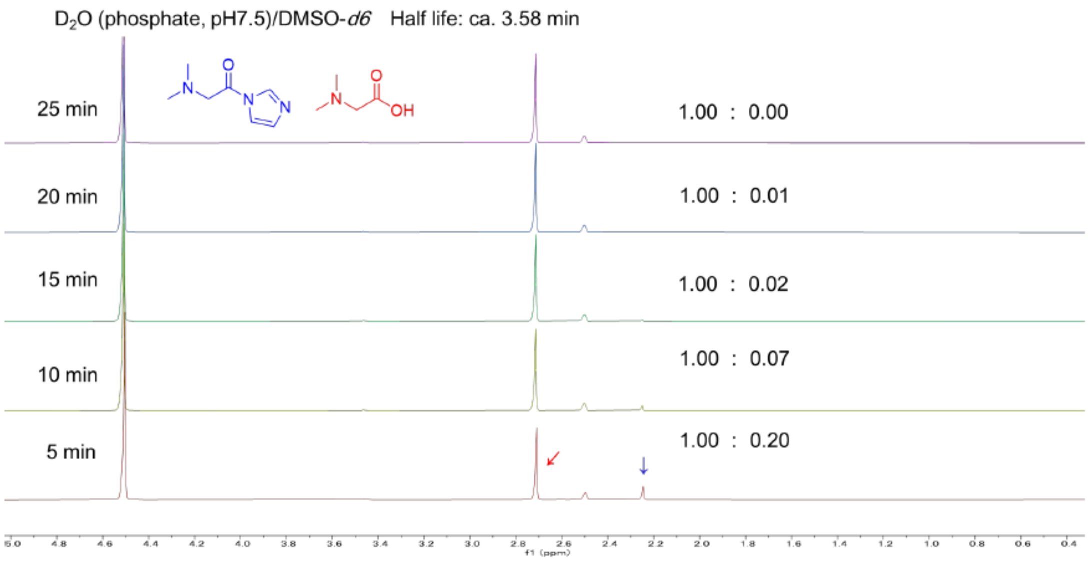
D$_2$O (phosphate, pH7.5)/DMSO-*d6* Half life: ca. 3.58 min
25 min 1.00 : 0.00
20 min 1.00 : 0.01
15 min 1.00 : 0.02
10 min 1.00 : 0.07
5 min 1.00 : 0.20
f1 (ppm)

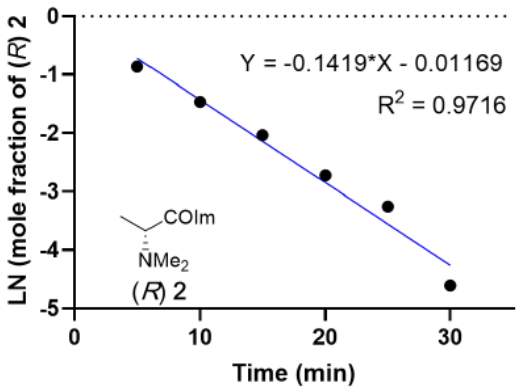
Y = -0.1419*X - 0.01169
R^2 = 0.9716
COIm
NMe$_2$
(*R*) 2
LN (mole fraction of (*R*) 2
Time (min)

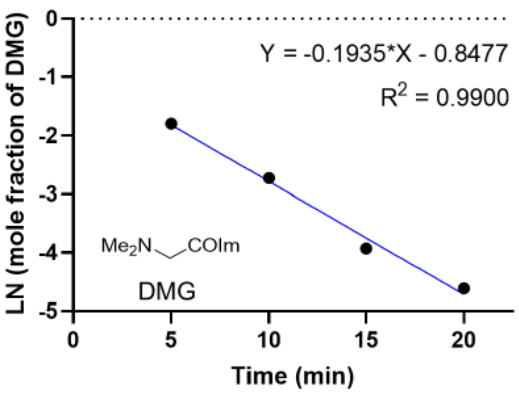
Y = -0.1935*X - 0.8477
R^2 = 0.9900
Me$_2$N COIm
DMG
LN (mole fraction of DMG)
Time (min)

Figure S3. Time courses for hydrolysis of N,N-dimethylalanine acylimidazole ((*R*)-**2**, top, and N,N-dimethylglycine (DMG, bottom)) as measured by NMR at 23°C in deuterated aqueous buffer (pD=7.5). Estimated half-lives are 3~4min (DMG) and 4~5min ((*R*)-**2**).

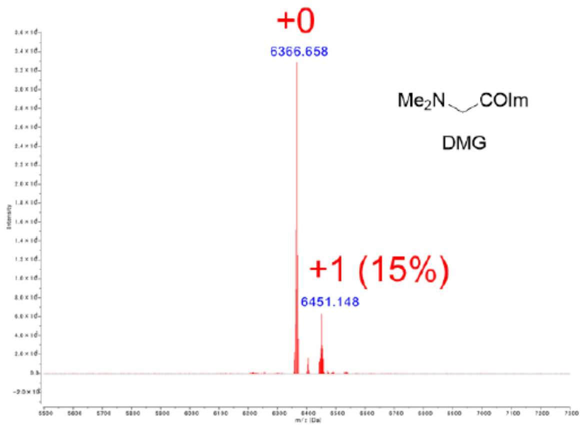

Figure S4. Reactivity of dimethylglycine with ssRNA as measured by mass spectrometry, showing 15% conversion with 100 μM reagent after 2 h (0°C). Same conditions for (*R*)-**2** yield 98% conversion (see Figure 2). ssRNA sequence is 5'- ACAAUUAUCCUAUGAGCGGU -3'. Conditions: 10 μM RNA, 100 μM reagent, MOPS buffer (100 mM) pH 7.5, 0 °C.

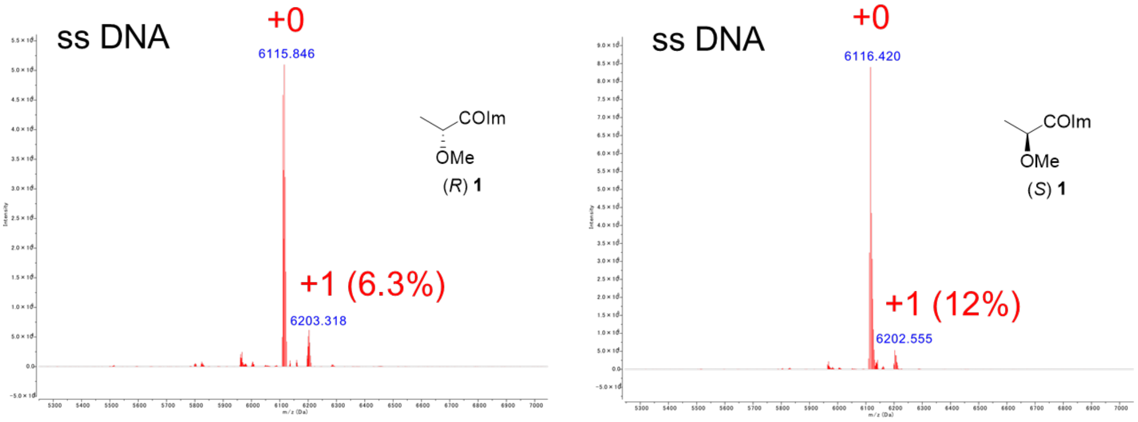

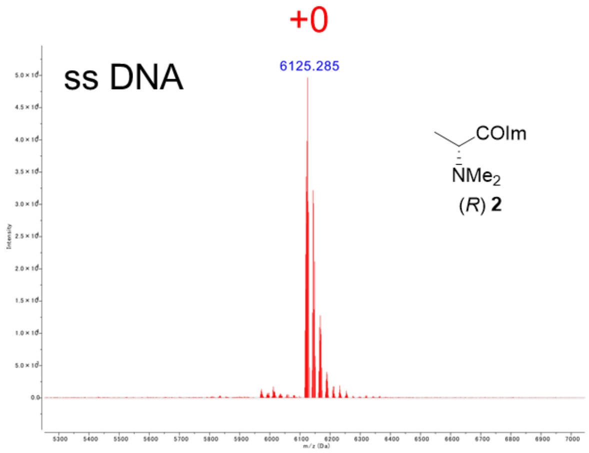
+0
ss DNA
6125.285
COIm
NMe2
(R) 2

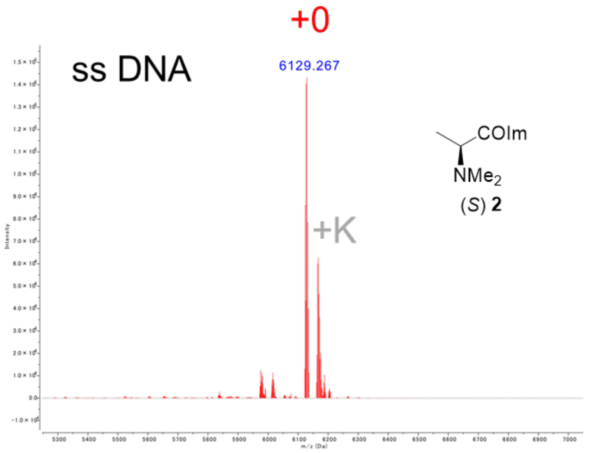
+0
ss DNA
6129.267
COIm
NMe2
(S) 2
+K

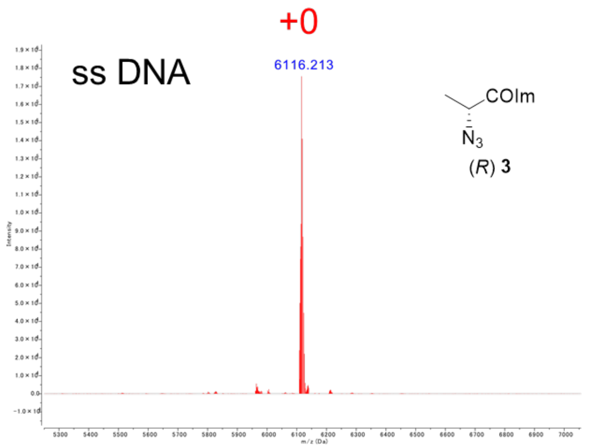
+0
ss DNA
6116.213
COIm
N3
(R) 3

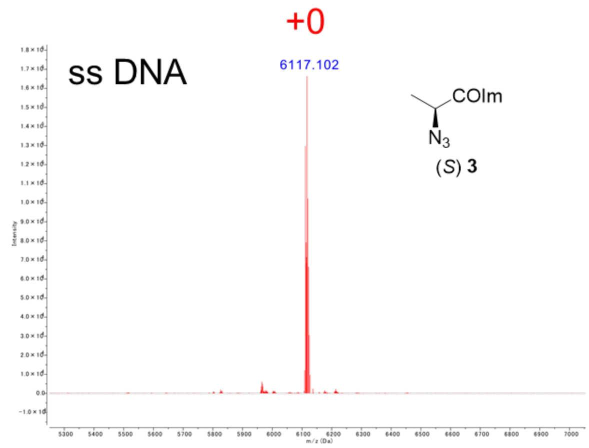
+0
ss DNA
6117.102
COIm
N3
(S) 3

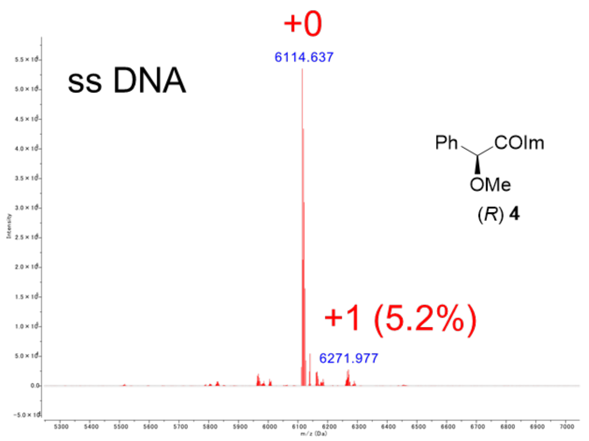
+0
ss DNA
6114.637
Ph
COIm
OMe
(R) 4
+1 (5.2%)
6271.977

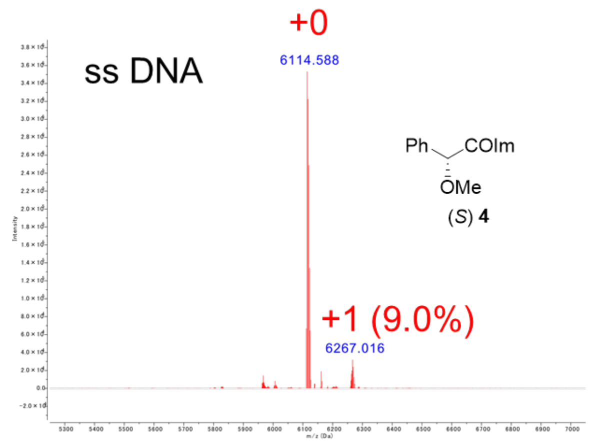
+0
ss DNA
6114.588
Ph
COIm
OMe
(S) 4
+1 (9.0%)
6267.016

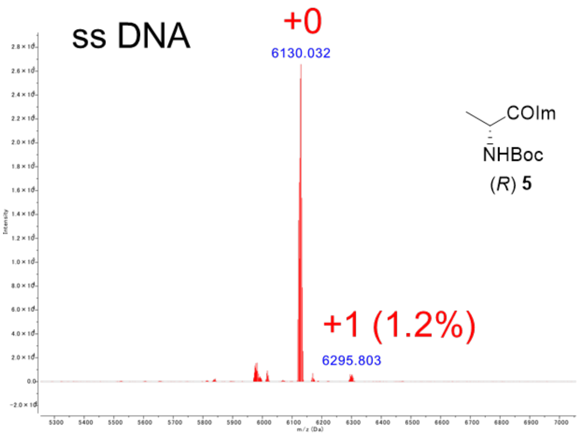
ss DNA
+0
6130.032
COIm
NHBoc
(R) 5
+1 (1.2%)
6295.803

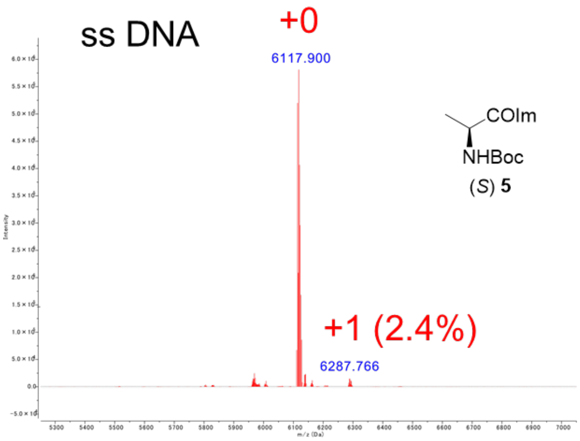
ss DNA
+0
6117.900
COIm
NHBoc
(S) 5
+1 (2.4%)
6287.766

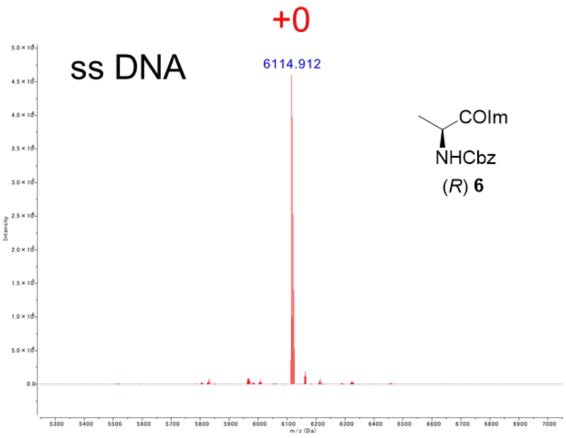
ss DNA
+0
6114.912
COIm
NHCbz
(R) 6

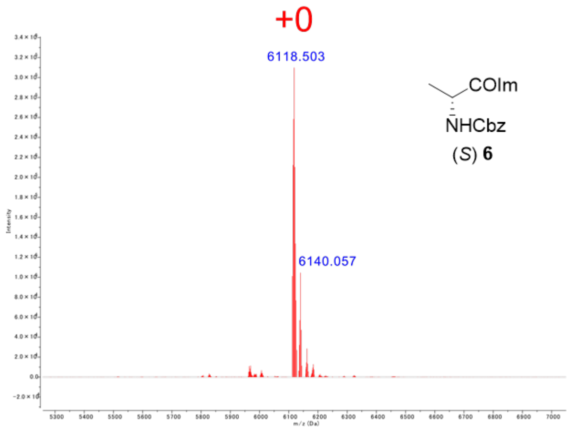
+0
6118.503
COIm
NHCbz
(S) 6
6140.057

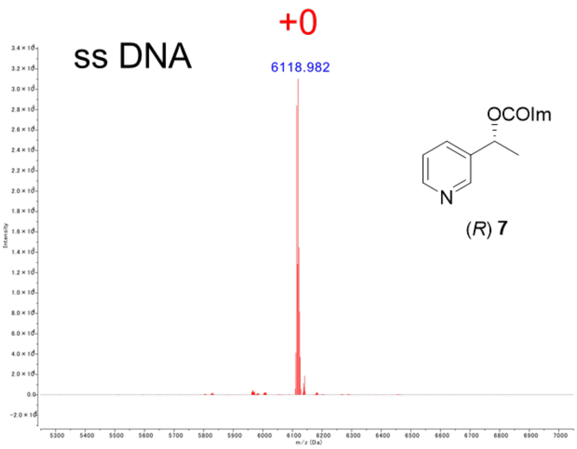
ss DNA
+0
6118.982
OCOIm
N
(R) 7

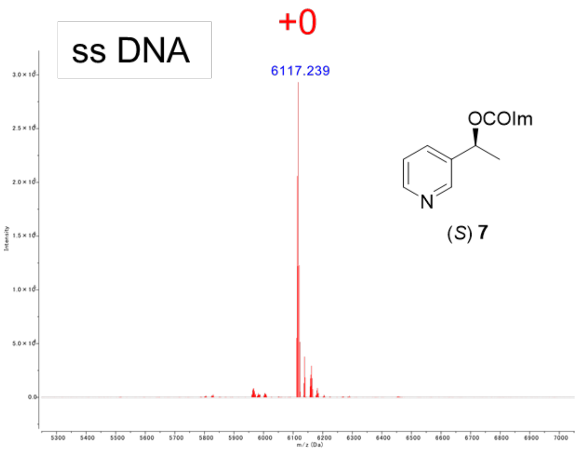
ss DNA
+0
6117.239
OCOIm
N
(S) 7

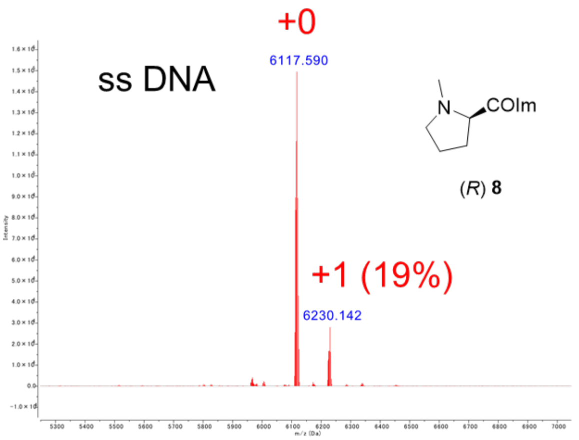
ss DNA
+0
6117.590
+1 (19%)
6230.142
COIm
(R) 8

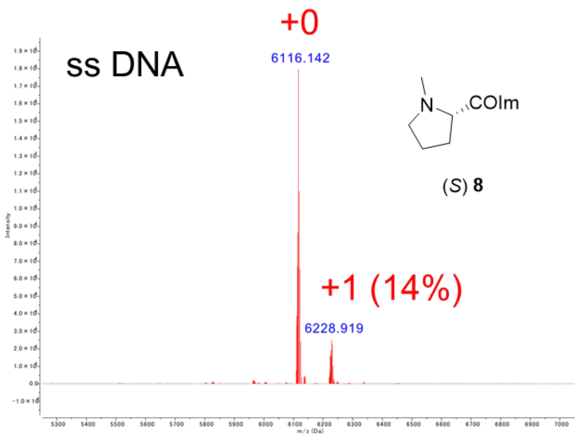
ss DNA
+0
6116.142
+1 (14%)
6228.919
COIm
(S) 8

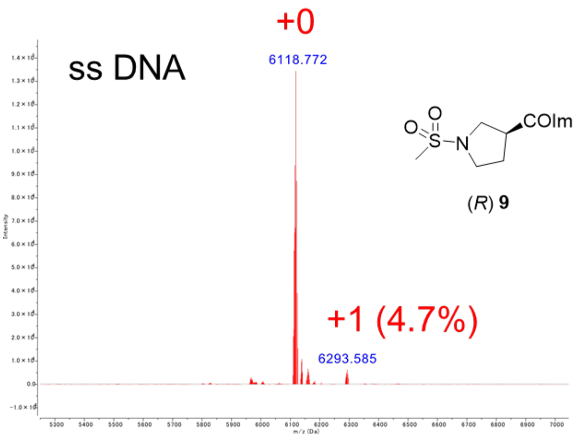
ss DNA
+0
6118.772
+1 (4.7%)
6293.585
COIm
(R) 9

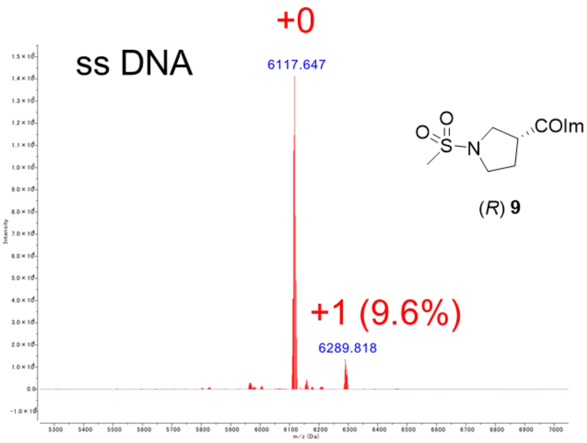
ss DNA
+0
6117.647
+1 (9.6%)
6289.818
COIm
(R) 9

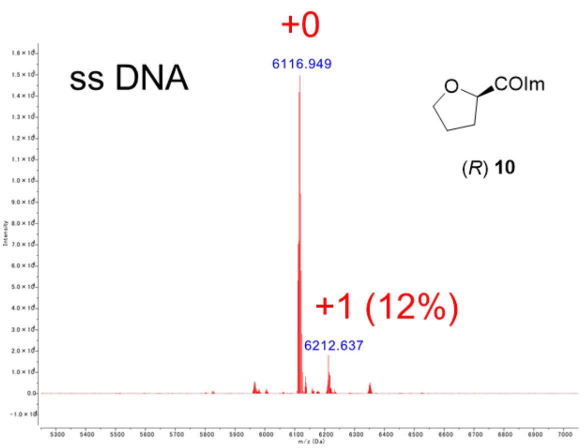
ss DNA
+0
6116.949
+1 (12%)
6212.637
COIm
(R) 10

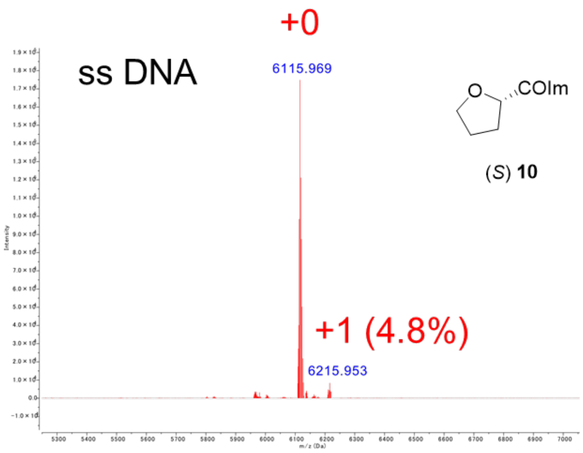
ss DNA
+0
6115.969
+1 (4.8%)
6215.953
COIm
(S) 10

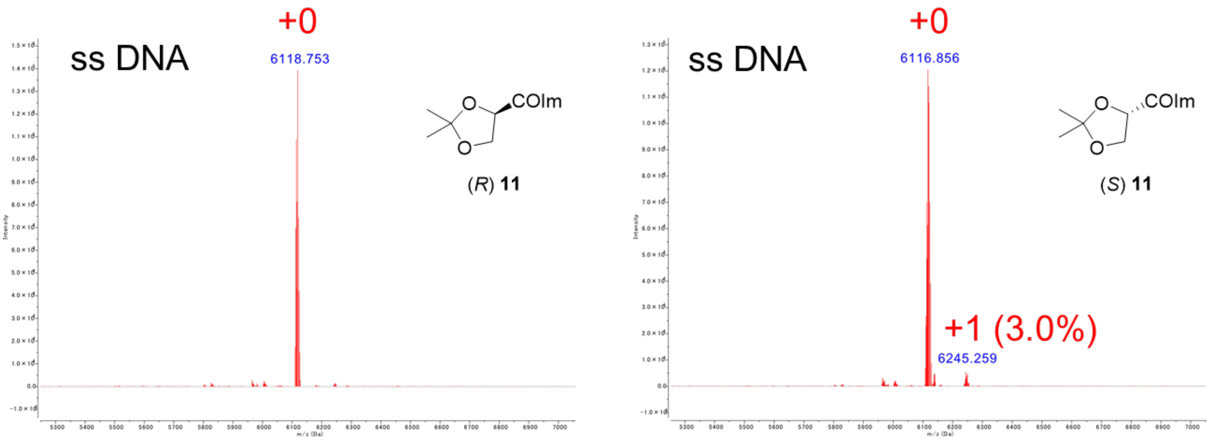

Figure S5. MALDI-TOF MS of DNA reactions for chiral acylating agents, showing near- or complete lack of reaction. The DNA is a single-stranded 18mer of the same sequence as the RNA in Fig. S1. The data confirm primary reaction at 2'-OH groups of RNA and not exocyclic amine groups. Conditions: 10 μM RNA, 200 mM reagent for (*R*), (*S*) **1**, **3**, **4**, **5**, **6**, **7**, **8**, **9**, **10**, **11** and 1 mM for (*R*), (*S*) **2**, MOPS buffer (100 mM) pH 7.5, 0 °C.

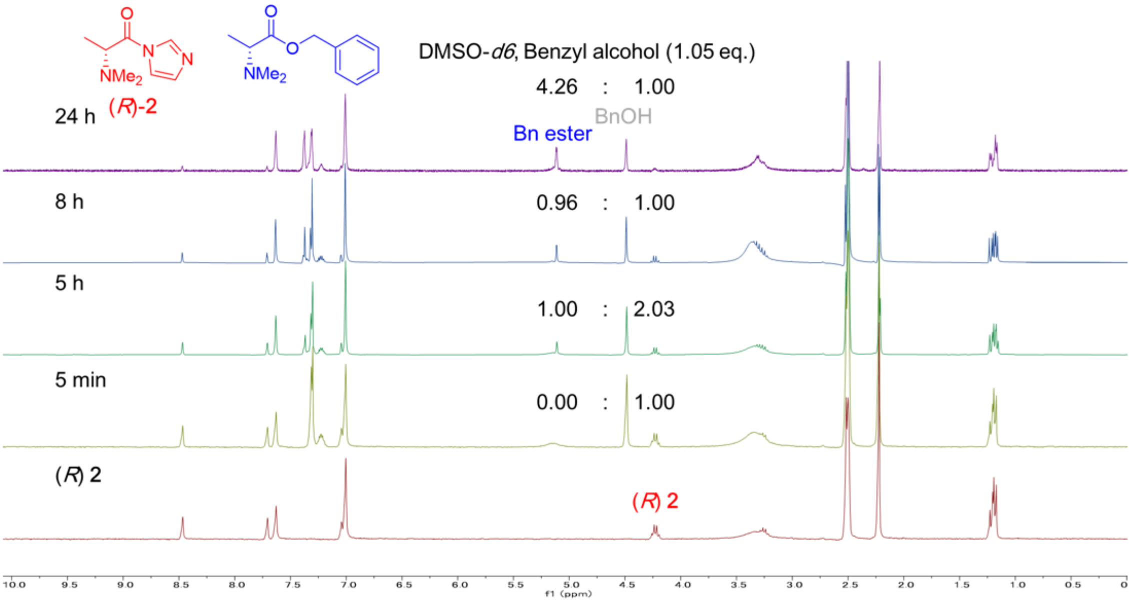

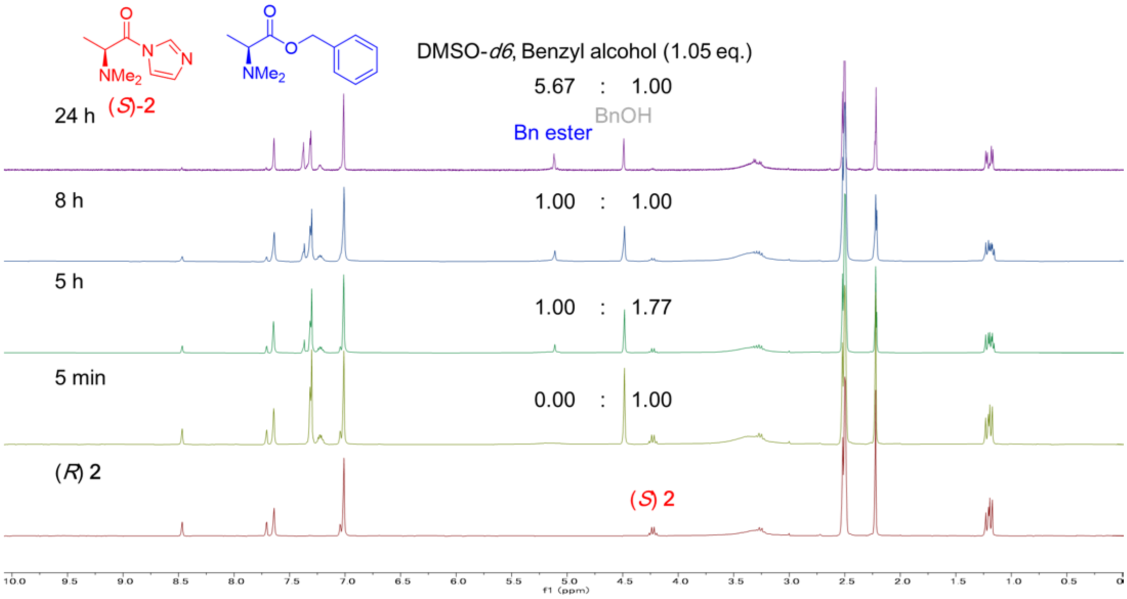

Figure S6. Confirmation of equal reactivity of (*R*)-**2** and (*S*)–**2** by reaction with an achiral alcohol (benzyl alcohol). Performed in NMR tube at room temperature, 1.05 eq benzyl alcohol in DMSO-d6. At top are NMR spectra; below is plot of conversions over time.

a

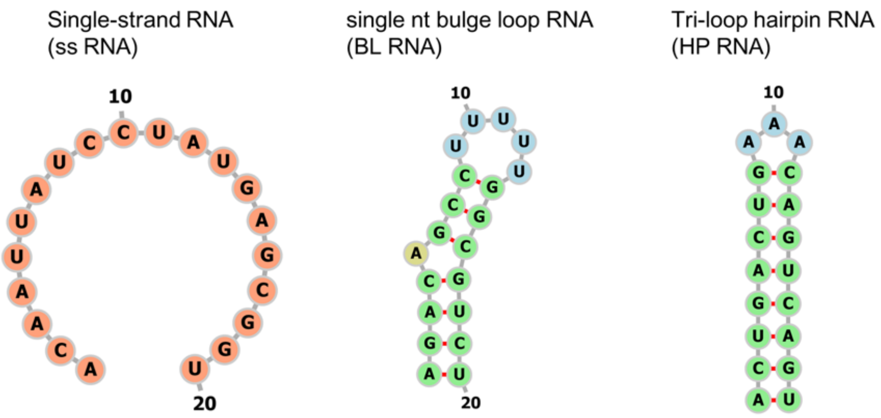

b

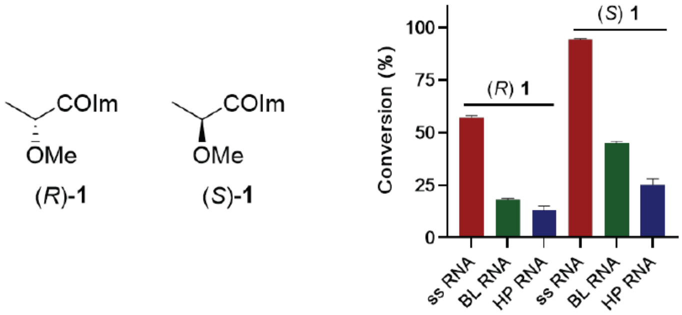

c

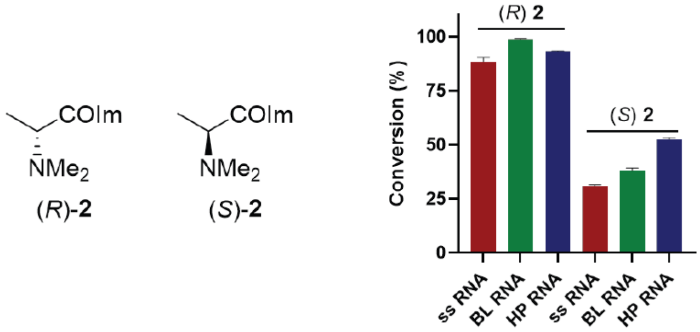

Figure S7. Diastereoselectivity as a function of structure for three RNAs as measured by MALDI-TOF MS. Shown are reaction conversions for three ssRNA, BL RNA, HP RNA) for acylation by (R,S)-1 and (R,S)-2. ssRNA sequence is 5'- ACAAUUAUCCUAUGAGCGGU -3'. (a) predicted structures of BL RNA and HP RNA. (b) Reaction conversions for (*R,S*)-**1**, showing lower yields for more highly structured RNAs as expected. (c) Reaction conversions for (*R,S*)-**2**. Data are averages of three reactions; error bars show standard deviations. Conditions: 10 µM RNA, 100 µM reagent for ss RNA, 250 µM reagent for BL and 1 mM reagent for HP RNA, MOPS buffer (100 mM) pH 7.5, 0 °C.

Table S1. Testing chiral reagent reactivity with single-stranded and structured RNAs. Reaction conversions of RNAs are shown.[a, b]

compound	ssRNA (%)[c]	hairpin (%)[d]	bulge loop (%)[e]
(R) **1**	57	18	13
(S) **1**	94	45	25
(R) **2**	89	99	93
(S) **2**	31	38	53

[a]Conversion of starting RNA to acylated products as measured by MALDI-TOF MS. Values are averages of three separate experiments.
[b]Conditions: 10 µM RNA, reagent at concentrations below, 20% DMSO in 100mM MOPS buffer (pH 7.5), 100mM NaCl, 6mM $MgCl_2$, 2 h, 0 °C.
[c]Treated with 80mM reagent for (*R,S*)-**1** and 100µM for (*R,S*)-**2**.
[d]Treated with 50mM reagent for (*R,S*)-**1** and 250µM for (*R,S*)-**2**.
[e]Treated with 100mM reagent for (*R,S*)-**1** and 1mM for (*R,S*)-**2**.

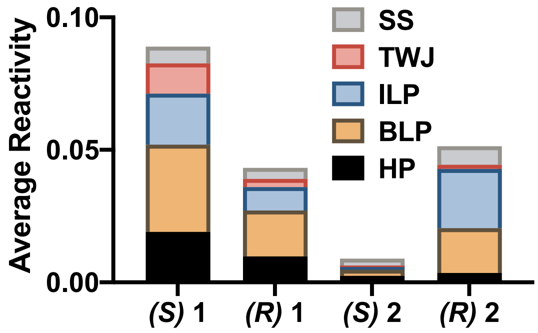

Figure S8. Average reactivity comparison of RNA motifs across different enantiomers (*R,S*)-**1** and (*R,S*)-**2**. Bulge loops and internal loops are the most reactive contexts for the chiral reagents.

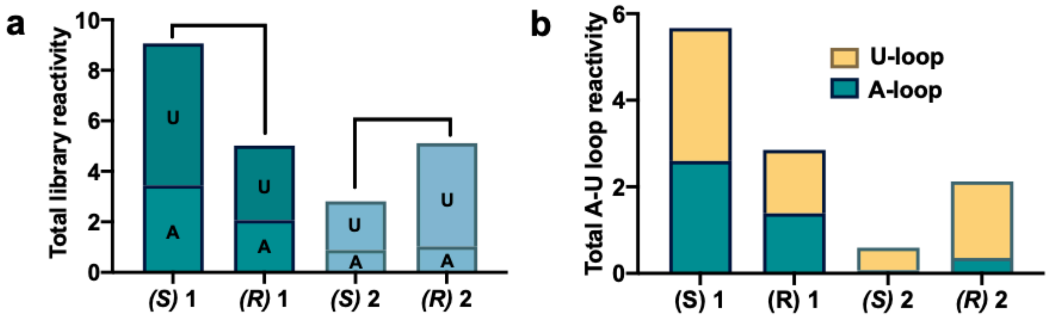

Figure S9. (a) Total library reactivity of two pairs of enantiomers (*R,S*)-**1** and (*R,S*)-**2**. For compounds **1**, (*S)* conformation is more reactive than (*R*), which is opposite to compounds **2**. (b) Total loop reactivity in A-case library vs. U-case library. Uridine loops are generally more reactive than those composed of adenosine nucleotides.

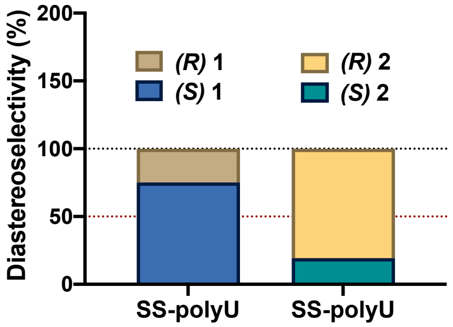

Figure S10. Diastereoselectivity of two pairs of enantiomers (*R,S*)-**1** and (*R,S*)-**2** for polyuridine single-stranded RNA (SS-polyU). The results show significant stereoselectivity for both chiral reagents **1** and **2** to SS-polyU.

	A-case		U-case	
	(S) 2	*(R)* 2	*(S)* 2	*(R)* 2
H4-1	0.238	0.000	0.177	0.667
H4-2	1.006	0.552	0.452	2.591
H4-3	0.558	0.035	0.035	1.273
H4-4	0.461	0.235	0.618	3.088
H4-5	0.146	0.391	0.000	7.607
H4-6	0.000	0.326	0.164	9.774
H4-7	0.349	1.199	0.593	1.483
H4-8	0.102	0.038	0.009	0.453
H4-9	0.022	0.038	0.000	0.092
H4-10	0.000	0.038	0.038	0.089
H4-11	0.000	0.000	0.000	0.000
B1-1	4.364	0.000	24.176	32.806
B1-2	261.505	184.706	13.604	11.986
B1-3	30.871	24.923	15.660	43.911
B1-4	4.737	25.851	6.773	54.359
B1-5	0.000	0.000	1.846	188.858
B1-6	0.000	0.000	0.000	59.845
B1-7	0.000	0.000	0.000	0.000
B1-8	0.000	0.000	10.708	3.536
B1-9	0.000	0.000	79.329	3.993
B1-10	0.000	0.000	3.446	6.304
B1-11	0.000	0.000	0.765	3.489
B1-12	0.946	0.760	0.190	1.718
B1-13	0.000	0.000	0.508	4.934
B1-14	0.097	0.466	0.000	0.837
B1-15	0.113	0.085	0.071	0.679
B1-16	0.000	0.000	0.876	0.627
B1-17	0.000	0.000	0.124	1.220
B1-18	0.000	0.000	0.000	0.000

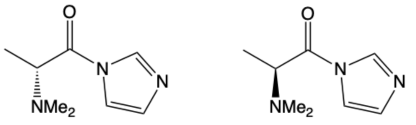

(*S*) 2 (*R*) 2

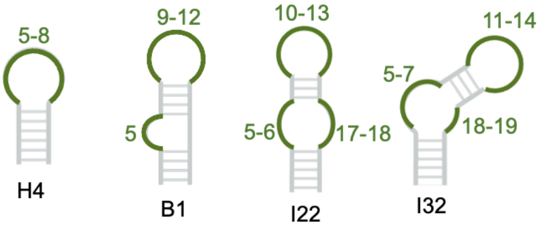

	A-case		U-case	
	(S) 2	*(R)* 2	*(S)* 2	*(R)* 2
I22-1	19.851	0.000	2.932	7.512
I22-2	12.276	0.000	43.360	44.909
I22-3	40.305	34.723	8.722	5.885
I22-4	13.618	44.183	10.475	64.983
I22-5	5.899	19.411	0.036	145.534
I22-6	0.000	111.291	0.000	58.820
I22-7	0.000	0.000	0.000	0.317
I22-8	1.724	0.000	2.194	2.555
I22-9	1.377	0.000	12.318	6.281
I22-10	0.078	0.000	14.562	9.631
I22-11	0.247	0.000	9.335	10.642
I22-12	0.190	0.000	1.795	5.026
I22-13	0.718	0.016	0.000	6.159
I22-14	0.211	0.000	1.458	5.853
I22-15	0.127	0.076	0.000	1.999
I22-16	0.990	0.000	4.448	2.487
I22-17	0.564	1.631	0.723	1.441
I22-18	0.000	0.042	1.774	1.908
I22-19	0.000	0.000	1.191	2.436
I22-20	0.016	0.000	0.004	0.090
I22-21	0.000	0.000	0.000	0.000
I32-1	16.804	0.000	49.838	22.065
I32-2	46.067	23.428	9.096	3.935
I32-3	38.199	64.979	8.881	24.897
I32-4	2.695	35.258	24.765	106.564
I32-5	1.776	11.644	9.776	214.798
I32-6	1.665	212.978	0.000	83.868
I32-7	0.085	0.000	0.000	0.000
I32-8	0.000	0.000	0.000	0.000
I32-9	5.399	0.000	0.000	0.421
I32-10	2.292	0.000	10.372	1.019
I32-11	1.147	0.000	7.997	6.578
I32-12	1.133	0.000	6.948	4.809
I32-13	2.503	0.000	2.270	3.480
I32-14	2.160	0.000	0.000	0.853
I32-15	0.000	0.000	2.000	4.891
I32-16	0.809	0.561	0.378	1.680
I32-17	0.933	0.339	0.432	0.280
I32-18	0.856	0.024	0.646	1.471
I32-19	0.112	0.212	0.002	0.336
I32-20	0.047	0.000	0.005	0.025
I32-21	0.022	0.015	0.176	0.189
I32-22	0.000	0.000	0.000	0.000

Fig. S11. Second-order rate constants (10^{-3} $M^{-1}s^{-1}$) for 2'-OH acylation by (*S*)-**2** and (*R*)-**2** in a range of RNA loops and stems based on our profiling data. Loop nucleotides are shaded in green. The results show that the relative rates vary greatly with local structure, which appears to be the dominant factor in selectivity.

Calculation method:

$\ln [RNA]_t = -k' * t + \ln [RNA]_0$

$k' = k_2 * [reagent]$

t: reaction time; $[RNA]_t$: RNA concentration at t time; $[RNA]_0$: RNA original concentration; k': Pseudo-1st-order rate constant; k_2 : second-order rate constant; [reagent]: acylating reagent concentration

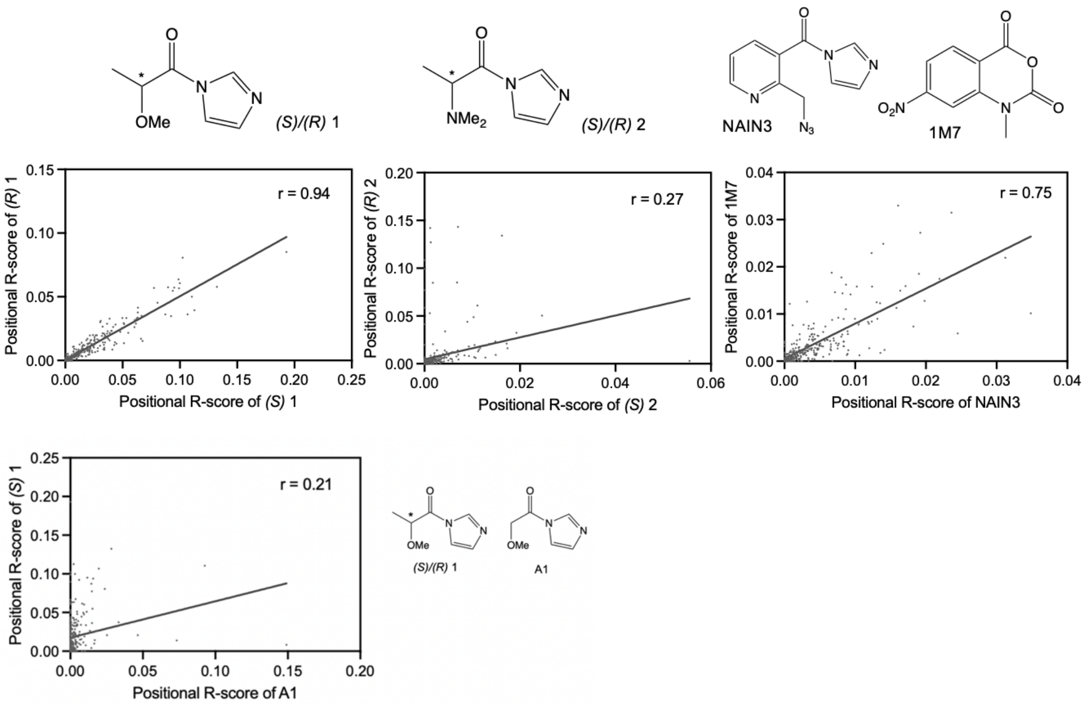

Fig. S12. Nucleotide-by-nucleotide positional correlation of reactivity of enantiomers of reagents **1** and **2** in loop nucleotides and single strands of RNA library. The results show very low correlation of enantiomers of the more stereoselective **2**, but substantially high positive correlation of enantiomers of **1**, which displays lower stereoselectivity. Also note lack of correlation in reactions of chiral (*S*)-**1** and achiral analog A1 (bottom). For comparison is shown correlation data for previous structurally similar achiral aromatic reagents NAIN3 and 1M7 studied in previous work (top right).[1]

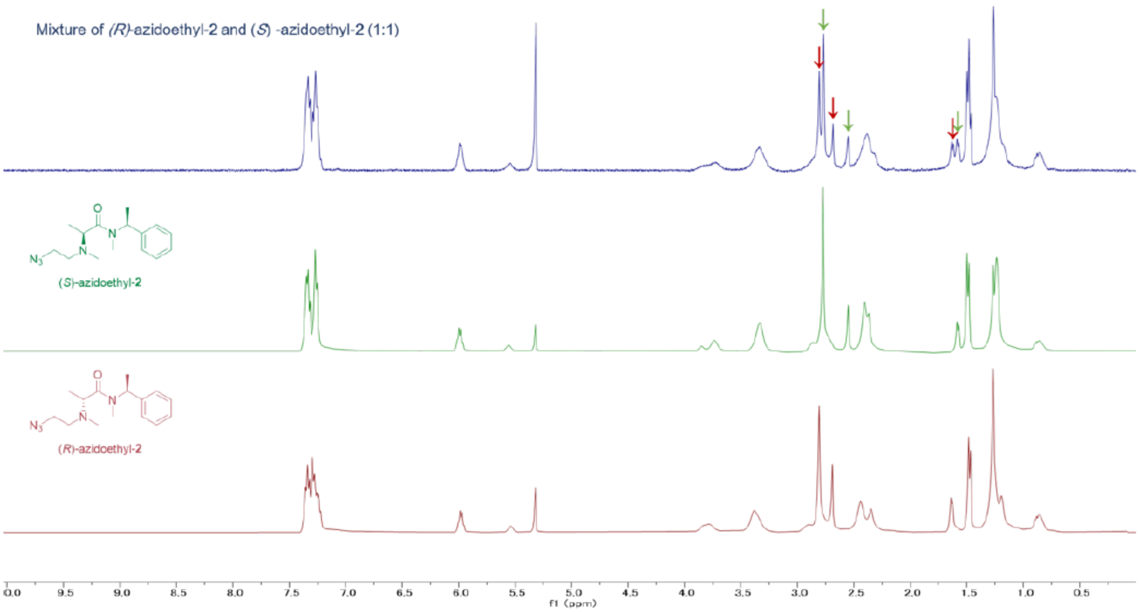

Figure S13. Confirmation of chiral purity (>90%) of azide-functionalized (*R,S*)-azidoethyl-**2** after synthesis, measured by derivatization with chiral (*S*) phenethylamine to form chemically distinct diastereomers (RS336,RS337). [1]NMR spectra are shown (δ 1-4 ppm, CD_2Cl_2). The data show that the synthesis steps required to add the azidoethyl group (*Scheme 1*) did not appreciably racemize the α-carbons that originated with the purchased starting materials.

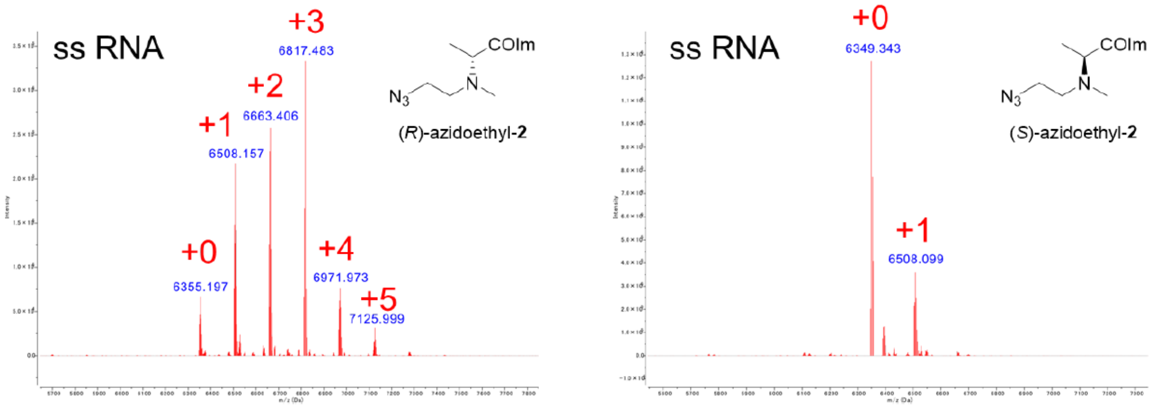

Figure S14. Diastereoselectivity of reaction of (*R,S*)-azidoethyl-**2** with ssRNA as measured by mass spectrometry. Red numbers (*e.g*, +1) show numbers of adducts per RNA strand. Conditions: 10 μM RNA, 2 mM reagent, MOPS (100 mM) buffer pH 7.5, 0 °C.

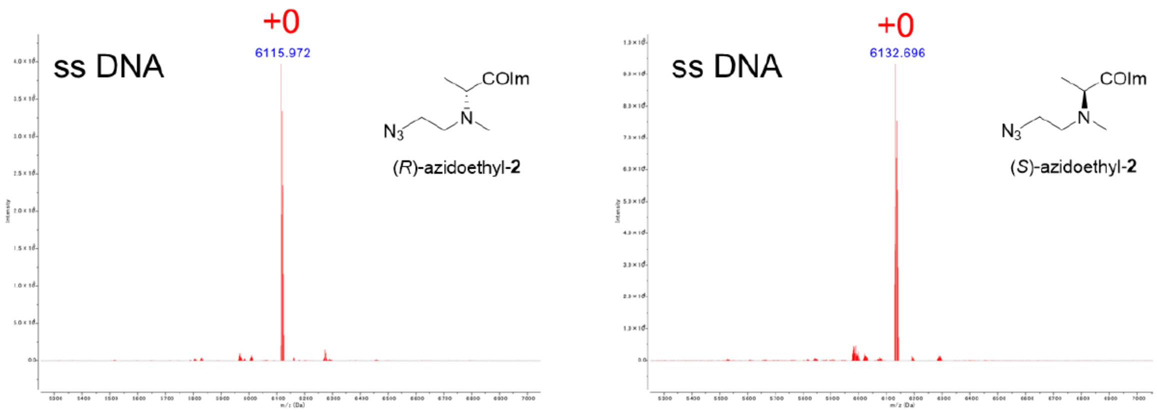

Figure S15. Lack of DNA reactions for (*R*)-azidoethyl-**2** and (*S*) -azidoethyl-**2**. Conditions: 10 µM DNA, 200 mM reagent, MOPS buffer (100 mM) pH 7.5, 0 °C.

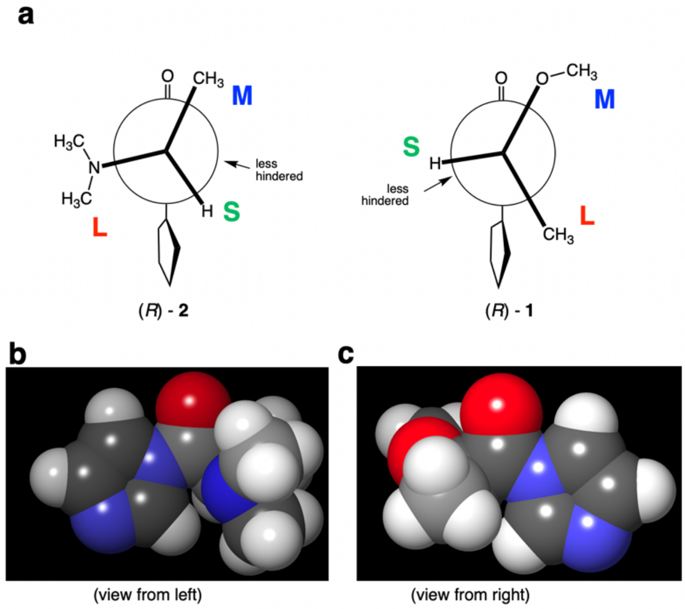

Figure S16. Conformational models proposed to explain observation that α-amino compounds (*e.g*. **2**,**8**) show opposite diastereoselectivity ((*R*) preferred) relative to α-alkoxy compounds (*e.g*. **1**,**10**; (*S*) preferred) in RNA 2'-OH acylations. (a) Newmann projections illustrating predicted low-energy conformers of (*R*)-**2** and (*R*)-**1** (OPLS forcefield in Macromodel (water solvent)). The dialkylamine group is sterically larger than the α-methyl group in **2**, whereas in **1**, the methyl group is sterically larger than the oxygen of the alkoxy group. In the model, the 2'-OH trajectory for reaction is governed by the least hindered facial approach to the carbonyl carbon. (b) Hindered face (left-side) view of space-filling model of (*R*)-**2** in conformation shown in (a), showing how dialkylamine partially blocks access to carbonyl carbon on that face. Shown is energy-minimized

structure (OPLS force field, Maestro application). (c) Hindered face (right side) view of space-filling model of (*R*)-**1** in conformation shown in (a), showing how methyl group marginally blocks access to carbonyl carbon on that face.

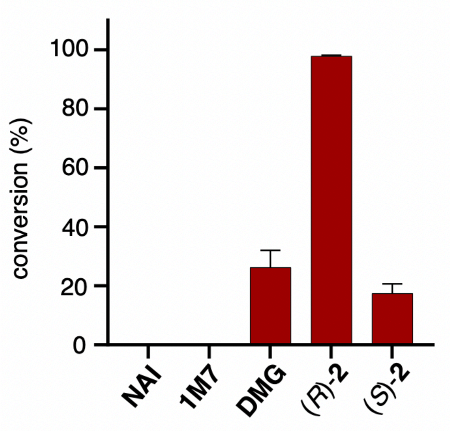

Figure S17. Benchmarking the RNA reactivity of new chiral reagents (*R*)-**2**, (*S*)-**2** against achiral reagent DMG (N,N-dimethylglycine) and two widely used acylating agents used for mapping RNA structure (NAI and 1M7). Reagents were used at 100 uM for 2h. Chiral reagent (*R*)-**2** is the most reactive RNA acylating agent reported to date. Conditions: MOPS (100 mM, pH 7.5) containing $MgCl_2$ (6mM), NaCl (100mM), 0°C.

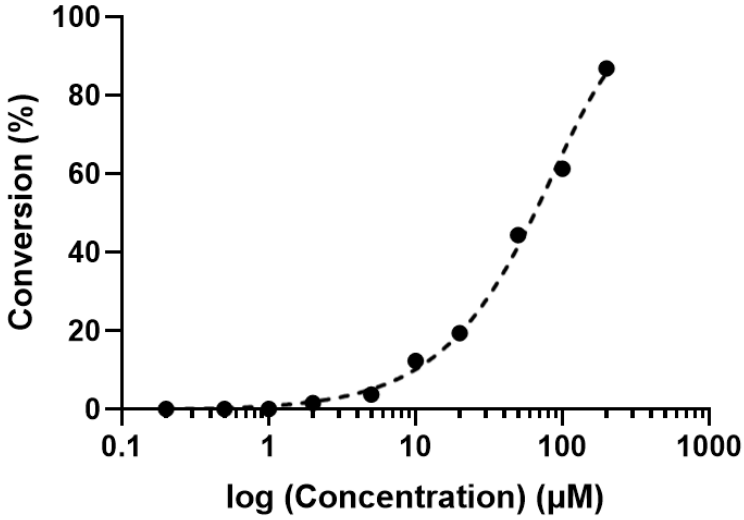

Figure S15. Effect of reagent concentration on reaction conversion with single-stranded RNA for highly reactive chiral compound (*R*)-**2**. Conditions: [RNA] 10 μM, reagent *R*-**2** added as concentrated stocks in DMSO, reaction time 2 h, 0°C in pH 7.5 buffer (100 mM MOPS) containing 100 mM Na+, 6.7 mM Mg2+, 20% DMSO, total volume 10 μL each reaction. Conversions are the yield of modified RNA divided by unmodified starting RNA as measured by MALDI-TOF mass spectrometry.

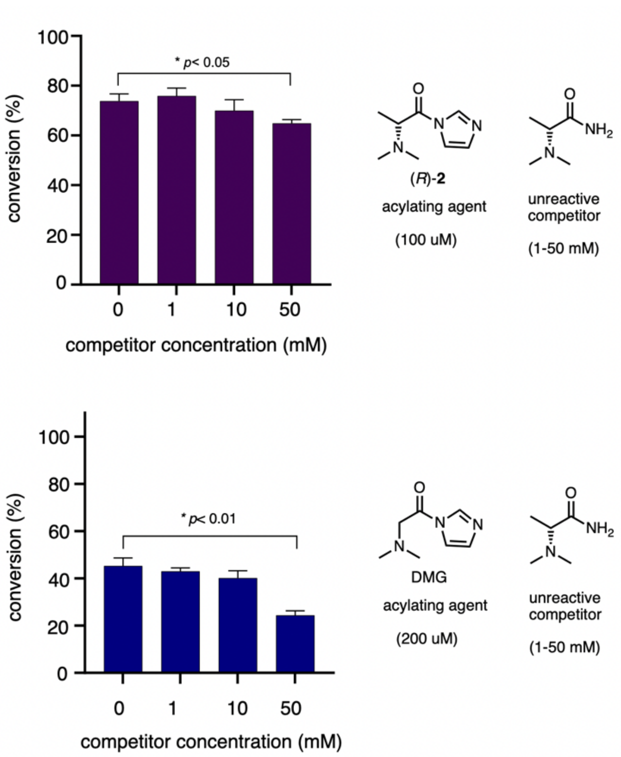

Figure S19. Effect of chiral competitor on reactivity of reagent (*R*)-**2** and achiral reagent DMG with single-stranded RNA. Effects are only seen at the highest competitor concentrations, and are similar for both a chiral highly reactive species and an achiral less reactive one. The data suggest that any binding by (*R*)-**2** is likely to be nonselective. This is also supported by the fact that (*R*)-**2** reacts similarly at several loop types with varied structure (Fig. 3b, main text).

Synthetic procedures

General procedure for the preparation of acylimidazoles

To a solution of carboxylic acid (1 equivalent) in anhydrous DMSO (as 1 M stock solution) was added CDI (1.5-1.7 equivalent) at ambient temperature under argon. The reaction mixture was stirred at ambient temperature 1~3 hours to afford the acylated reagent as mixture with imidazole. For NMR analysis, the reactions were performed in DMSO-*d6*.

(*S*)-1-(1H-Imidazol-1-yl)-2-methoxypropan-1-one ((*S*)-**1**)

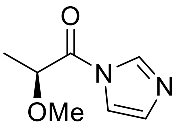

Analytical data:

¹H NMR (300 MHz, DMSO-*d6*) δ 8.48 (s, 1H), 7.71 (s, 1H), 7.11 (s, 1H), 4.77 (q, *J* = 6.3 Hz, 1H), 3.32 (s, 3H), 1.39 (d, *J* = 6.1 Hz, 3H)

¹³C NMR (75 MHz, DMSO-*d6*) δ 170.09, 137.17, 130.38, 116.60, 75.65, 56.58, 17.57

ESI-MS [M+H]: Calculated: 155.08; Observed: 154.99

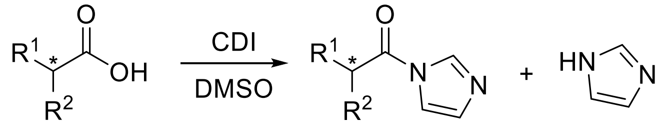

(*R*)-1-(1H-Imidazol-1-yl)-2-methoxypropan-1-one ((*R*)-**1**)

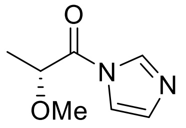

Analytical data:

¹H NMR (500 MHz, DMSO-*d6*) δ 8.48 (s, 1H), 7.75 (s, 1H), 7.11 (s, 1H), 4.78 (q, *J* = 6.7 Hz, 1H), 3.32 (s, 3H), 1.39 (d, *J* = 6.6 Hz, 3H)

¹³C NMR (125 MHz, DMSO-*d6*) δ 170.10, 137.17, 130.36, 116.60, 75.67, 56.58, 17.55

ESI-MS [M+H]: Calculated: 155.08; Observed: 155.07

(*S*)-2-(Dimethylamino)-1-(1H-imidazol-1-yl)propan-1-one ((*S*)-**1**)

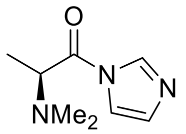

Analytical data:

¹H NMR (500 MHz, DMSO-*d6*) δ 8.48 (s, 1H), 7.71 (s, 1H), 7.05 (s, 1H), 4.22 (q, *J* = 6.4 Hz, 1H), 2.22 (s, 6zH), 1.18 (d, *J* = 6.7 Hz, 3H)

¹³C NMR (125 MHz, DMSO-*d6*) δ 170.24, 137.66, 129.85, 116.72, 60.87, 40.42, 8.68

ESI-MS [M+H]: Calculated: 168.11; Observed: 168.10

(*R*)-2-(Dimethylamino)-1-(1H-imidazol-1-yl)propan-1-one ((*R*)-**2**)

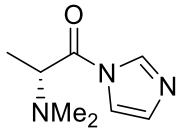

Analytical data:

¹H NMR (500 MHz, DMSO-*d6*) δ 8.48 (s, 1H), 7.71 (s, 1H), 7.05 (s, 1H), 4.22 (q, *J* = 6.7 Hz, 1H), 2.22 (s, 6zH), 1.18 (d, *J* = 6.7 Hz, 3H)

¹³C NMR (125 MHz, DMSO-*d6*) δ 170.23, 137.66, 129.85, 116.72, 60.85, 40.43, 8.68

ESI-MS [M+H]: Calculated: 168.11; Observed: 168.10

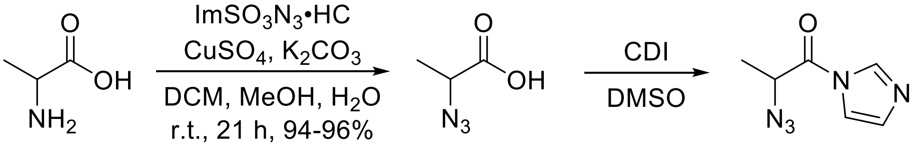

(*R*)-**12** (*R*)-**13** (*R*)-**3**
(*S*)-**12** (*S*)-**13** (*S*)-**3**

(*S*)-2-Azidopropanoic acid ((*S*)-**12**)

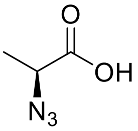

Compound **13** was obtained following the reported procedure.[4]

^{1}H NMR (300 MHz, Chloroform-*d*) δ 4.03 (q, *J* = 7.0 Hz, 1H), 1.54 (d, *J* = 7.2 Hz, 3H)

Analytical data were found to be identical to literature data.[5]

(*S*)-2-Azido-1-(1H-imidazol-1-yl)propan-1-one ((*S*)-**3**)

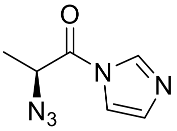

Analytical data:

^{1}H NMR (300 MHz, DMSO-*d6*) δ 8.50 (1H, s), 7.77 (s, 1H), 7.13 (s, 1H), 5.09 (q, *J* = 6.8 Hz, 1H), 1.50 (d, *J* = 7.0 Hz, 3H)

^{13}C NMR (75 MHz, DMSO-*d6*) δ 168.44, 137.45, 130.76, 116.84, 56.44, 16.49.

ESI-MS [M+H]: Calculated: 166.07; Observed: 166.16

(*R*)-2-Azidopropanoic acid ((*R*)-**12**)

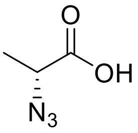

Compound **12** was obtained following reported procedure.[4]

^{1}H NMR (300 MHz, Chloroform-*d*) δ 4.03 (q, *J* = 7.2 Hz, 1H), 1.54 (d, *J* = 7.2 Hz, 3H)

Analytical data were found to be identical to literature data.[5]

(*R*)-2-Azido-1-(1H-imidazol-1-yl)propan-1-one ((*R*)-**3**)

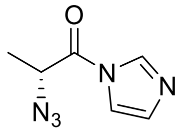

Analytical data:

^{1}H NMR (300 MHz, DMSO-*d6*) δ 8.50 (1H, s), 7.77 (s, 1H), 7.13 (s, 1H), 5.09 (q, *J* = 6.9 Hz, 1H), 1.50 (d, *J* = 6.9 Hz, 3H)

^{13}C NMR (75 MHz, DMSO-*d6*) δ 168.44, 137.46, 130.76, 116.86, 56.45, 16.50.

ESI-MS [M+H]: Calculated: 166.07; Observed: 166.20

(*S*)-1-(1H-Imidazol-1-yl)-2-methoxy-2-phenylethan-1-one ((*S*)-**4**)

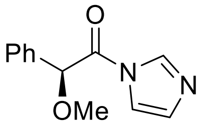

Analytical data:

^{1}H NMR (300 MHz, DMSO-*d*) δ 8.55 (s, 1H), 7.76 (s, 1H), 7.51 (d, *J* = 7.7 Hz, 2H), 7.43-7.36 (m, 3H), 7.06 (s, 1H), 5.86 (s, 1H), 3.39 (s, 3H)

^{13}C NMR (75 MHz, DMSO-*d*) δ 167.87, 137.18, 130.45, 129.19, 128.94, 127.73, 116.77, 81.37, 57.04

ESI-MS [M+H]: Calculated: 217.09; Observed: 217.11

(*R*)-1-(1H-Imidazol-1-yl)-2-methoxy-2-phenylethan-1-one ((*R*)-**4**)

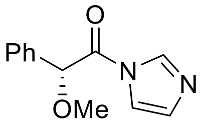

Analytical data:

^{1}H NMR (300 MHz, DMSO-*d*) δ 8.55 (s, 1H), 7.76 (s, 1H), 7.51 (d, *J* = 7.7 Hz, 2H), 7.43-7.36 (m, 3H), 7.06 (s, 1H), 5.86 (s, 1H), 3.39 (s, 3H)

^{13}C NMR (75 MHz, DMSO-*d*) δ 167.86, 137.17, 130.44, 129.18, 128.93, 127.72, 116.76, 81.35, 57.03

ESI-MS [M+H]: Calculated: 217.09; Observed: 217.10

tert-Butyl (*S*)-(1-(1H-imidazol-1-yl)-1-oxopropan-2-yl)carbamate ((*S*)-**5**)

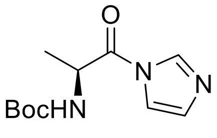

Analytical data:

^{1}H NMR (500 MHz, DMSO-*d*) δ 8.50 (s, 1H)), 7.76 (s, 1H), 7.10 (s, 1H), 4.94-4.88 (m, 1H), 1.36 (s, 9H), 1.33 (d, *J* = 7.2 1H)

^{13}C NMR (125 MHz, DMSO-*d*) δ 171.08, 155.37, 136.95, 130.40, 116.65, 78.78, 49.33, 28.08, 16.78

ESI-MS [M+H]: Calculated: 240.13; Observed: 240.11

tert-Butyl (*R*)-(1-(1H-imidazol-1-yl)-1-oxopropan-2-yl)carbamate ((*R*)-**5**)

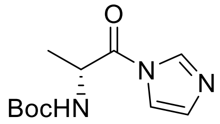

Analytical data:

^{1}H NMR (500 MHz, DMSO-*d*) δ 8.50 (s, 1H)), 7.76 (s, 1H), 7.10 (s, 1H), 4.93-4.88 (m, 1H), 1.36 (s, 9H), 1.32 (d, *J* = 6.8 1H)

^{13}C NMR (125 MHz, DMSO-*d*) δ 171.06, 155.35, 136.94, 130.39, 116.64, 78.77, 49.30, 28.07, 16.77

ESI-MS [M+H]: Calculated: 274.11; Observed: 274.20

Benzyl (*S*)-(1-(1H-imidazol-1-yl)-1-oxopropan-2-yl)carbamate ((*S*)-**6**)

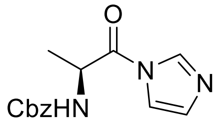

Analytical data:

^{1}H NMR (500 MHz, DMSO-*d*) δ 8.53 (s, 1H), 8.21 (d, *J* = 6.3 Hz, 1H), 7.78 (s, 1H), 7.37-7.30 (m, 4H), 7.12 (s, 1H), 5.05 (s, 2H), 5.03-4.99 (m, 1H), 1.38 (dd, *J* = 6.9, 2.9 Hz, 3H).

^{13}C NMR (125 MHz, DMSO-*d*) δ 170.81, 155.96, 137.01, 136.75, 130.53, 128.39, 127.93, 127.76, 116.69, 65.83, 49.67, 16.91

ESI-MS [M+H]: Calculated: 274.11; Observed: 274.20

Benzyl (*R*)-(1-(1H-imidazol-1-yl)-1-oxopropan-2-yl)carbamate ((*R*)-**6**)

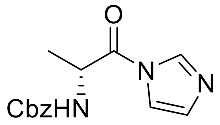

Analytical data:

^{1}H NMR (500 MHz, DMSO-*d*) δ 8.52 (s, 1H), 8.18 (s, 1H), 7.77 (s, 1H), 7.37-7.30 (m, 4H), 7.12 (s, 1H), 5.05 (s, 2H), 5.02-4.97 (m, 1H), 1.36 (d, *J* = 7.2 Hz, 3H).

^{13}C NMR (125 MHz, DMSO-*d*) δ 170.78, 155.92, 136.98, 136.73, 130.51, 128.36, 127.90, 127.74, 116.66, 65.79, 49.63, 16.88

ESI-MS [M+H]: Calculated: 274.11; Observed: 274.25

(*S*)-1-(Pyridin-3-yl)ethyl 1H-imidazole-1-carboxylate ((*S*)-**7**)

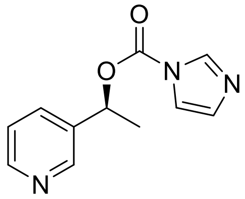

Analytical data:

^{1}H NMR (300 MHz, DMSO-*d*) δ 8.75 (s, 1H), 8.55 (d, *J* = 2.8 Hz, 1H), 8.35 (s, 1H), 7.95 (d, *J* = 8.1 Hz, 1H), 7.44-7.40 (m, 1H), 7.08 (s, 1H), 6.10 (q, *J* = 6.5 Hz, 1H), 1.67 (d, *J* = 6.6 Hz, 1H).

^{13}C NMR (75 MHz, DMSO-*d*) δ 149.60, 147.81, 147.64, 137.41, 135.94, 130.38, 123.73, 117.59, 74.69, 21.64.

ESI-MS [M+H]: Calculated: 218.09; Observed: 218.11

(*R*)-1-(Pyridin-3-yl)ethyl 1H-imidazole-1-carboxylate ((*R*)-**7**)

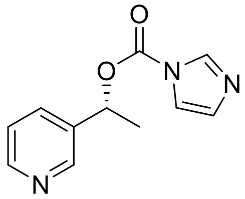

Analytical data:

^{1}H NMR (300 MHz, DMSO-*d*) δ 8.74 (d, *J* = 1.9 Hz, 1H), 8.55 (dd, *J* = 4.8, 1.6 Hz, 1H), 8.35 (s, 1H), 7.95 (d, *J* = 8.3 Hz, 1H), 7.44-7.40 (m, 1H), 7.08 (s, 1H), 6.10 (q, *J* = 6.1 Hz, 1H), 1.67 (d, *J* = 7.2 Hz, 1H).

^{z13}C NMR (75 MHz, DMSO-*d*) δ 149.59, 147.79, 147.63, 137.40, 135.76, 130.37, 123.73, 117.58, 74.69, 21.63.

ESI-MS [M+H]: Calculated: 218.09; Observed: 218.12

1-(Methyl-D-prolyl)-1H-imidazole ((*R*)-**8**)

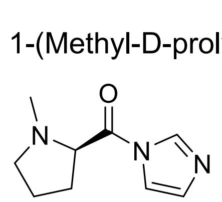

Analytical data:

^{1}H NMR (400 MHz, DMSO-*d*) δ 8.53 (s, 1H), 7.76 (s, 1H), 7.08 (s, 1H), 3.79 (dd, *J* = 8.6, 5.7 Hz, 1H), 3.03 (dd, *J* = 8.1, 5.7 Hz, 1H), 2.41-2.30 (m, 5H), 1.87-1.79 (m, 2H).

^{13}C NMR (75 MHz, DMSO-*d*) δ 170.54, 137.06, 130.19, 116.66, 67.02, 55.43, 40.06, 29.98, 23.16.

ESI-MS [M+H]: Calculated: 180.11; Observed: 180.16

1-(Methyl-L-prolyl)-1H-imidazole ((*S*)-**8**)

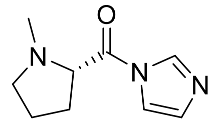

To a solution of activated molecular sieves 4A in toluene was added 1-Methyl-L-proline of half solid in small amount of methanol, stirred at ambient temperature for 1 hour. The mixture was concentrated in vacuo and the precipitate was filtered, washed with hexane. The residue was dried in high vacuum to afford the white solid. The white solid was used in the next general procedure without further purification.

^{1}H NMR (400 MHz, DMSO-*d*) δ 8.54 (s, 1H), 7.76 (s, 1H), 7.09 (s, 1H), 3.79 (dd, *J* = 9.1, 6.2 Hz, 1H), 3.03 (dd, *J* = 8.2, 5.2 Hz, 1H), 2.41-2.30 (m, 5H), 1.89-1.77 (m, 2H).

^{13}C NMR (100 MHz, DMSO-*d*) δ 170.58, 137.10, 130.21, 116.69, 67.08, 55.46, 40.08, 30.01, 23.18.

ESI-MS [M+H]: Calculated: 180.11; Observed: 180.17

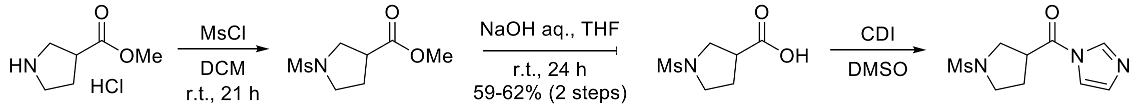

Methyl (*S*)-1-(methylsulfonyl)pyrrolidine-3-carboxylate ((*S*)-**15)**

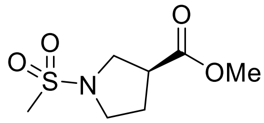

To a solution of (*S*)-Methyl Pyrrolidine-3-carboxylate hydrochloride ((*S*)-**14**) (300 mg, 2.32 mmol) in anhydrous dichloromethane (9.2 mL) was added triethylamine (680 µL, 4.88 mmol) and Methanesulfonyl chloride (216 µL, 2.79 mmol) at ambient temperature. The reaction mixture was stirred at ambient temperature for 1 hours under argon. After the reaction, the mixture was diluted with dichloromethane and washed with water. The organic layer was dried over anhydrous sodium sulfate, filtered and concentrated in vacuo to afford the crude (*S*)-**15** as a pale brown oil.

^{1}H NMR (300 MHz, CDCl3) δ 3.75 (s, 3H), 3.67-3.35 (m, 4H), 3.20-3.15 (m, 1H), 2.87 (s, 3H), 2.34-2.22 (m, 2H).

Analytical data were found to be identical to literature data.[6]

(*S*)-1-(Methylsulfonyl)pyrrolidine-3-carboxylic acid ((*S*)-**16)**

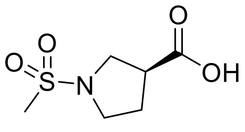

To a solution of crude (*S*)-**15** in tetrahydrofuran (10 mL) was added 1N NaOH aq. (4.7 mL) at ambient temperature. The reaction mixture was stirred at ambient temperature for 24 hours under argon. After the reaction, the mixture was quenched with 1N HCl aq. and t extracted with dicholoromethane 2 times. The organic layer was dried over anhydrous sodium sulfate, filtered and concentrated in vacuo. The residue was eluted with small amount of chloroform and diluted with hexane, and store -20℃ over night. The precipitate was collected to afford the (*S*)-**16** as a white solid.

^{1}H NMR (300 MHz, DMSO-*d*) δ 3.46-3.23 (m, 4H), 3.15-3.05 (m, 1H), 2.88 (s, 3H), 2.18-1.97 (m, 2H).

Analytical data were found to be identical to literature data.[3]

(*S*)-(1H-Imidazol-1-yl)(1-(methylsulfonyl)pyrrolidin-3-yl)methanone ((*S*)-**9**)

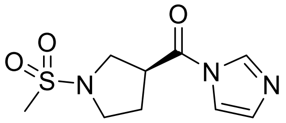

Analytical data:

^{1}H NMR (500 MHz, DMSO-*d*) δ 8.53 (s, 1H), 7.78 (s, 1H), 7.11 (s, 1H), 4.07 (q, *J* = 6.8, 1H), 3.63 (dd, *J* = 9.5, 8.1, 1H), 3.52 (dd, *J* = 10. 6.3, 1H) 3.37-3.33 (m, 2H), 2.38-2.32 (m, 1H), 2.17-2.10 (m, 1H).

^{13}C NMR (125 MHz, DMSO-*d*) δ 170.33, 137.26, 130.57, 116.74, 49.38, 47.24, 42.66, 33.72, 29.27.

ESI-MS [M+H]: Calculated: 244.07; Observed: 244.10

Methyl (*R*)-1-(methylsulfonyl)pyrrolidine-3-carboxylate ((*R*)-**15)**

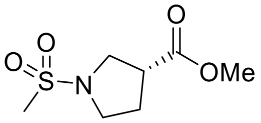

To a solution of (*R*)-Methyl Pyrrolidine-3-carboxylate hydrochloride ((*R*)-**14**) (300 mg, 2.32 mmol) in anhydrous dichloromethane (9.2 mL) was added triethylamine (680 μL, 4.88 mmol) and Methanesulfonyl chloride (216 μL, 2.79 mmol) at ambient temperature. The reaction mixture was stirred at ambient temperature for 1 hours

under argon. After the reaction, the mixture was diluted with dichloromethane and washed with water. The organic layer was dried over anhydrous sodium sulfate, filtered and concentrated in vacuo to afford the crude (*R*)-**15** as a pale brown oil.

^{1}H NMR (300 MHz, CDCl3) δ 3.73 (s, 3H), 3.67-3.35 (m, 4H), 3.17-3.13 (m, 1H), 2.86 (s, 3H), 2.27-2.17 (m, 2H).

Analytical data were found to be identical to literature data.[6]

(*R*)-1-(Methylsulfonyl)pyrrolidine-3-carboxylic acid ((*R*)-**16**)

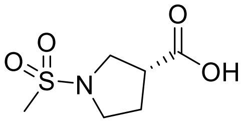

To a solution of crude (*R*)-**16** in tetrahydrofurane (10 mL) was added 1N NaOH aq. (4.7 mL) at ambient temperature. The reaction mixture was stirred at ambient temperature for 24 hours under argon. After the reaction, the mixture was quenched with 1N HCl aq. and extracted with dicholoromethane 2 times. The organic layer was dried over anhydrous sodium sulfate, filtered and concentrated in vacuo. The residue was eluted with small amount of chloroform and diluted with hexane, and store -20°C over night. The precipitate was collected to afford the crude (*S*)-**16** as a white solid.

^{1}H NMR (300 MHz, DMSO-*d*) δ 3.47-3.24 (m, 4H), 3.15-3.06 (m, 1H), 2.89 (s, 3H), 2.18-1.97 (m, 2H).

Analytical data were found to be identical to literature data.[6]

(*R*)-(1H-Imidazol-1-yl)(1-(methylsulfonyl)pyrrolidin-3-yl)methanone ((*R*)-**9**)

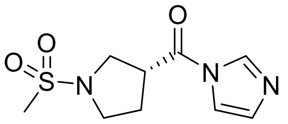

Analytical data:

^{1}H NMR (500 MHz, DMSO-*d*) δ 8.53 (s, 1H), 7.78 (s, 1H), 7.11 (s, 1H), 4.07 (q, *J* = 7.2, 1H), 3.63 (dd, *J* = 9.8, 7.4, 1H), 3.52 (dd, *J* = 10. 6.2, 1H) 3.36-3.33 (m, 2H), 2.39-2.32 (m, 1H), 2.17-2.10 (m, 1H).

^{13}C NMR (125 MHz, DMSO-*d*) δ 170.33, 137.26, 130.57, 116.74, 49.38, 47.24, 42.66, 33.72, 29.27.

ESI-MS [M+H]: Calculated: 244.07; Observed: 244.11

(*R*)-(1H-Imidazol-1-yl)(tetrahydrofuran-2-yl)methanone ((*R*)-**10**)

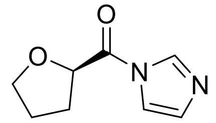

Analytical data:

^{1}H NMR (300 MHz, DMSO-*d*) δ 8.45 (s, 1H), 7.72 (s, 1H), 7.10 (s, 1H), 5.24 (dd, *J* = 7.8, 5.1 Hz, 1H), 3.86 (dd, *J* = 6.6, 6.6 Hz, 1H), 2.35-2.23 (m, 1H), 2.15-2.04 (m, 1H), 1.94-1.84 (m, 2H).

^{13}C NMR (75 MHz, DMSO-*d*) δ 169.85, 137.28, 130.27, 116.66, 76.56, 69.03, 28.99, 25.14.

ESI-MS [M+H]: Calculated: 167.08; Observed: 167.13

(*S*)-(1H-Imidazol-1-yl)(tetrahydrofuran-2-yl)methanone ((*S*)-**10**)

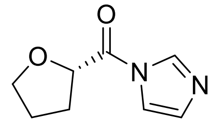

Analytical data:

δ 8.45 (s, 1H), 7.71 (s, 1H), 7.09 (s, 1H), 5.23 (dd, *J* = 8.1, 5.2 Hz, 1H), 3.86 (dd, *J* = 6.9, 6.9 Hz, 1H), 2.32-2.23 (m, 1H), 2.14-2.03 (m, 1H), 1.93-1.84 (m, 2H).

^{13}C NMR (75 MHz, DMSO-*d*) δ 169.85, 137.28, 130.28, 116.66, 76.56, 69.03, 28.99, 25.15.

ESI-MS [M+H]: Calculated: 167.08; Observed: 167.06

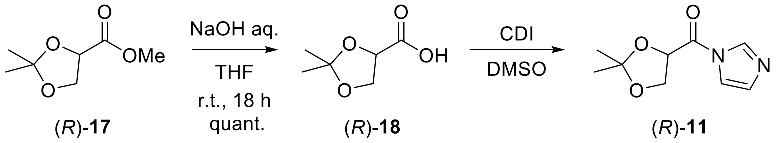

(*R*)-2,2-Dimethyl-1,3-dioxolane-4-carboxylic acid ((*R*)-**18**)

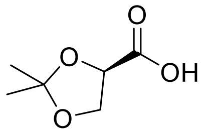

Compound xx was obtained following reported procedure.[7]

^{1}H NMR (300 MHz, DMSO-*d*) δ 4.60 (dd, *J* = 7.5, 4.8 Hz, 1H), 4.30 (dd, *J* =8.9, 7.6 Hz, 1H), 4.18 (dd, *J* = 8.7, 4.8 Hz, 1H), 1.54 (s, 3H), 1.42 (s, 3H).

Analytical data were found to be fully consistent with literature data.[7]

(*R*)-(2,2-Dimethyl-1,3-dioxolan-4-yl)(1H-imidazol-1-yl)methanone ((*R*)-**11**)

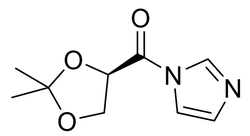

General procedure

^{1}H NMR (300 MHz, DMSO-*d*) δ 8.45 (s, 1H), 7.73 (s, 1H), 7.11 (s, 1H), 5.45 (dd, *J* = 6.6, 4.5 Hz, 1H), 4.32 (dd, *J* = 8.5, 7.1 Hz, 1H), 4.24 (dd, *J* = 8.8, 4.5 Hz, 1H), 1.38 (s, 6H).

^{13}C NMR (75 MHz, DMSO-*d*) δ 167.98, 137.25, 130.40, 116.64, 110.86, 74.15, 65.94, 25.70, 25.18.

ESI-MS [M+H]: Calculated: 197.09; Observed: 197.11

(*S*)-2,2-Dimethyl-1,3-dioxolane-4-carboxylic acid ((*S*)-**18**)

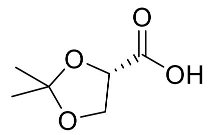

Compound xx was obtained following reported procedure.[7]

^{1}H NMR (300 MHz, DMSO-*d*) δ 4.61 (dd, *J* = 7.4, 4.7 Hz, 1H), 4.30 (dd, *J* =8.6, 7.3 Hz, 1H), 4.18 (dd, *J* = 8.9, 4.8 Hz, 1H), 1.54 (s, 3H), 1.42 (s, 3H).

Analytical data were found to be fully consistent with literature data.[7]

(*S*)-(2,2-Dimethyl-1,3-dioxolan-4-yl)(1H-imidazol-1-yl)methanone ((*S*)-**11**)

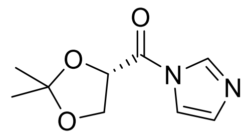

General procedure

^{1}H NMR (300 MHz, DMSO-*d*) δ 8.45 (s, 1H), 7.74 (s, 1H), 7.11 (s, 1H), 5.45 (dd, *J* = 7.3, 4.5 Hz, 1H), 4.32 (dd, *J* = 8.5, 7.2 Hz, 1H), 4.24 (dd, *J* = 8.7, 4.5 Hz, 1H), 1.38 (s, 6H).

^{13}C NMR (75 MHz, DMSO-*d*) δ 167.98, 137.25, 130.40, 116.64, 110.86, 74.15, 65.94, 25.71, 25.19.

ESI-MS [M+H]: Calculated: 197.09; Observed: 197.09

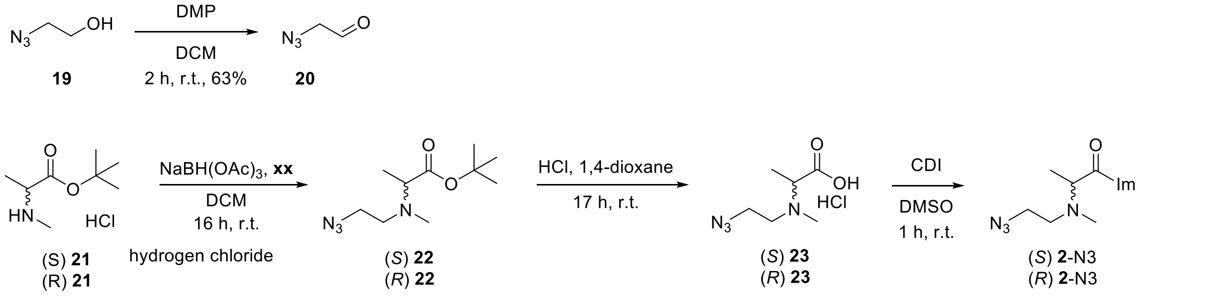

2-Azidoacetaldehyde (**20**)

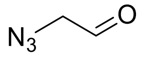

Compound **20** was obtained following reported procedure.[8] (Caution! Explosion hazard!)

^{1}H NMR (500 MHz, D_2O) δ 5.15 (t, *J* = 4.8 Hz, 1H), 3.33 (d, *J* = 4.8 Hz, 3H).

Analytical data were found to be fully consistent with literature data.[8]

tert-Butyl N-(2-azidoethyl)-N-methyl-L-alaninate ((*S*)-**22**)

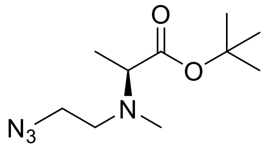

To a solution of *tert*-Butyl methyl-L-alaninate ((*S*)-**21**) (396 mg, 2.02 mmol) and Azidoacetaldehyde (190 mg, 2.23 mmol) in dichloromethane (20 mL) was added Sodium triacetoxyborohydride (1.29 g, 6.08 mmol) at 0°C. The reaction mixture was stirred at ambient temperature for 16 hours under argon. After the reaction, the mixture was quenched with saturated $NaHCO_3$ aq. and extracted with dicholoromethane, dried over anhydrous sodium sulfate, filtered and concentrated in vacuo. The residue was purified with flash clomatography (silica, 10:1 hexane/ethyl acetate) to afford afford the product (*S*)-**22** (273 mg, 1.20 mmol, 59%) as a pale yellow oil.

^{1}H NMR (400 MHz, DMSO-*d*) δ 3.36-3.26 (m, 3H), 2.82-2.75 (m, 1H), 2.71-2.65 (m, 1H), 2.29 (s, 3H), 1.42 (s, 9H), 1.14 (d, *J* = 7.1 Hz, 3H).

^{13}C NMR (75 MHz, DMSO-*d*) δ 171.85, 80.07, 61.44, 52.89, 48.15, 37.36, 27.83, 14.98.

ESI-MS [M+H]: Calculated: 229.16; Observed: 229.20

N-(2-Azidoethyl)-N-methyl-L-alanine ((*S*)-**23**)

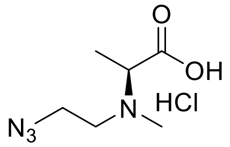

To a solution of (*S*)-**22** (205 mg, 0.90 mmol) in 4M 1,4-dioxane (13 mL) was stirred at ambient temperature for 17 hours under argon. After the reaction, the mixture was concentrated in vacuo. After the residue was purified with flash clomatography (silica, 3:1 dichloromethane/methanol), the pure product was eluted with mixture of solvent

(4:3:1 acetone:/dichloromethane/methanol) and filtererd. The fliltrate was concentrated and high vacuum over night to afford the product (*S*)-**23** (182 mg, 0.88 mmol, 98%) as a pale yellow oil.
^{1}H NMR (300 MHz, DMSO-*d*) δ 3.38 (q, *J* = 7.3 Hz, 1H), 3.32-3.28 (m, 2H), 2.85-2.67 (m, 2H), 2.31 (s, 3H), 1.18 (d, *J* = 7.1 Hz, 3H).
^{13}C NMR (75 MHz, DMSO-*d*) δ 173.87, 60.78, 52.84, 48.14, 37.46, 14.80.
ESI-MS [M+H]: Calculated: 173.10; Observed: 173.16

(*S*)-2-((2-Azidoethyl)(methyl)amino)-1-(1H-imidazol-1-yl)propan-1-one ((*S*)-**2** N3)

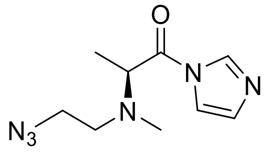

Analytical data
^{1}H NMR (400 MHz, DMSO-*d*) δ 8.50 (s, 1H), 7.73 (s, 1H), 7.05 (s, 1H), 4.41 (q, *J* = 6.7 Hz, 1H), 3.35-3.30 (m, 2H), 2.79-2.73 (m, 1H), 2.64-2.58 (m,1H), 2.26 (s, 3H), 1.20 (q, *J* = 6.6 Hz, 1H).
^{13}C NMR (100 MHz, DMSO-*d*) δ 170.34, 137.76, 129.88, 116.77, 60.66, 52.23, 48.16, 36.79, 9.41.
ESI-MS [M+H]: Calculated: 223.13; Observed: 223.20

tert-Butyl N-(2-azidoethyl)-N-methyl-D-alaninate ((*R*)-**22**)

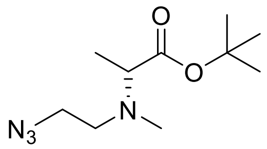

To a solution of *tert*-Butyl methyl-D-alaninate ((*R*)-**21**) (392 mg, 2.00 mmol) and Azidoacetaldehyde (188 mg, 2.21 mmol) in dichloromethane (20 mL) was added Sodium triacetoxyborohydride (1.27 g, 6.02 mmol) at 0°C. The reaction mixture was stirred at ambient temperature for 16 hours under argon. After the reaction, the mixture was quenched with saturated $NaHCO_3$ aq. and extracted with dicholoromethane, dried over anhydrous sodium sulfate, filtered and concentrated in vacuo. The residue was purified with flash clomatography (silica, 10:1 hexane/ethyl acetate) to afford the product (*R*)-**22** (273 mg, 1.20 mmol, 59%) as a pale ellow oil.
^{1}H NMR (500 MHz, DMSO-*d*) δ 3.35-3.27 (m, 3H), 2.81-2.76 (m, 1H), 2.71-2.66 (m, 1H), 2.29 (s, 3H), 1.41 (s, 9H), 1.14 (d, *J* = 7.2 Hz, 3H).
^{13}C NMR (75 MHz, DMSO-*d*) δ 171.84, 80.06, 61.44, 52.89, 48.16, 37.35, 27.83, 14.98.

ESI-MS [M+H]: Calculated: 229.16; Observed: 229.26

N-(2-Azidoethyl)-N-methyl-D-alanine ((*R*)-**23**)

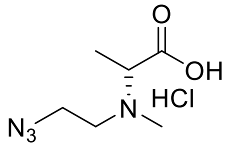

To a solution of (*R*)-**22** (257 mg, 1.13 mmol) in 4M 1,4-dioxane (13 mL) was stirred at ambient temperature for 17 hours under argon. After the reaction, the mixture was concentrated in vacuo. After the residue was purified with flash clomatography (silica, 3:1 dichloromethane/methanol), the pure product was eluted with mixture of solvent (4:3:1 acetone:/dichloromethane/methanol) and filtererd. The fliltrate was concentrated and high vacuum over night to afford afford the product (*R*)-**23** (203 mg, 0.98 mmol, 87%) as a pale yellow oil.

^{1}H NMR (300 MHz, DMSO-*d*) δ 3.95-3.88 (q, *J* = 7.0 Hz, 1H), 3.68-3.63 (m, 2H), 3.16-3.09 (m, 2H), 2.62 (s, 3H), 1.37 (d, *J* = 7.2 Hz, 3H)

^{13}C NMR (75 MHz, DMSO-*d*) δ 171.37, 61.06, 52.34, 46.59, 37.70, 13.00.

ESI-MS [M+H]: Calculated: 173.10; Observed: 173.14

(*R*)-2-((2-Azidoethyl)(methyl)amino)-1-(1H-imidazol-1-yl)propan-1-one ((*S*)-**2** N3)

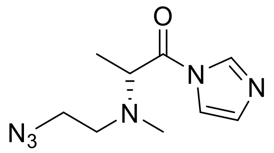

Analytical data:

^{1}H NMR (400 MHz, DMSO-*d*) δ 8.50 (s, 1H), 7.73 (s, 1H), 7.05 (s, 1H), 4.41 (q, *J* = 6.9 Hz, 1H), 3.34-3.30 (m, 2H), 2.79-2.73 (m, 1H), 2.64-2.58 (m,1H), 2.26 (s, 3H), 1.20 (q, *J* = 6.8 Hz, 1H).

^{13}C NMR (100 MHz, DMSO-*d*) δ 170.34, 137.75, 129.88, 116.76, 60.66, 52.23, 48.16, 36.79, 9.41.

ESI-MS [M+H]: Calculated: 223.13; Observed: 223.20

References

1) Xiao, L.; Fang, L.; Kool, E. T., Acylation probing of “generic” RNA libraries reveals critical influence of loop constraints on reactivity. *Cell Chem. Biol.* **2022**.

2) Flynn, R. A.; Zhang, Q. C.; Spitale, R. C.; Lee, B.; Mumbach, M. R.; Chang, H. Y., Transcriptome-wide interrogation of RNA secondary structure in living cells with icSHAPE. *Nat. Protoc.* **2016,** *11* (2), 273-290.
3) Chatterjee, S.; Shioi, R.; Kool, E. T. **2023**, manuscript in revision.
4) I. E. Valverde, A. Bauman, C. A. Kluba, S. Vomstein, M. A. Walter, T. L. Mindt *Angewandte Chemie, International Edition* **2013**, *52*, 8957-8960.
5) J. L. Lundquist, J. C. Pelletier *Organic Letters* **2001**, *3*, 781-783.
6) S. Jian, H. Lili, G. Yuanyu, Z. Lijuan, J. Jingwen, L. Yuanbai, J. Jinbo, M. Xueqin, M. Yangxiu, T. Dong, Y. Haikang, I. Zafar, Y. Zhixiang *Mendeleev Communications* **2021**, *31*, 498-500.
7) D. Iong, T.J. Church, J. R. Jacobsen, L. Jiang US patent WO2007/103187 A2
8) K. Ii, S. Ichikawa, B. Al-Dabbagh, A. Bouhss, A. Matsuda *Journal of Medicinal Chemistry* **2010**, *53*, 3793-3813.
